# A data-driven study of Alzheimer's disease related amyloid and tau pathology progression

**DOI:** 10.1093/brain/awad232

**Published:** 2023-07-11

**Authors:** Leon M Aksman, Neil P Oxtoby, Marzia A Scelsi, Peter A Wijeratne, Alexandra L Young, Isadora Lopes Alves, Lyduine E Collij, Jacob W Vogel, Frederik Barkhof, Daniel C Alexander, Andre Altmann, for the ADNI

**Affiliations:** Stevens Neuroimaging and Informatics Institute, Keck School of Medicine, University of Southern California, Los Angeles, CA 90033, USA; Centre for Medical Image Computing, Department of Medical Physics and Biomedical Engineering, University College London, London WC1V 6LJ, UK; Centre for Medical Image Computing, Department of Computer Science, University College London, London WC1V 6LJ, UK; Centre for Medical Image Computing, Department of Medical Physics and Biomedical Engineering, University College London, London WC1V 6LJ, UK; Centre for Medical Image Computing, Department of Computer Science, University College London, London WC1V 6LJ, UK; Department of Neuroimaging, Institute of Psychiatry, Psychology and Neuroscience, King’s College London, London SE5 8AF, UK; Centre for Medical Image Computing, Department of Computer Science, University College London, London WC1V 6LJ, UK; Brain Research Center, Amsterdam 1081 GN, The Netherlands; Department of Radiology and Nuclear Medicine, Amsterdam UMC, Vrije Universiteit Amsterdam, Amsterdam 1007MB, The Netherlands; Amsterdam Neuroscience, Brain Imaging, Amsterdam 1081 HV, The Netherlands; Department of Psychiatry, University of Pennsylvania, Philadelphia, PA 19104, USA; Lifespan Informatics and Neuroimaging Center, University of Pennsylvania, Philadelphia, PA 19104, USA; Centre for Medical Image Computing, Department of Medical Physics and Biomedical Engineering, University College London, London WC1V 6LJ, UK; Brain Research Center, Amsterdam 1081 GN, The Netherlands; Department of Radiology and Nuclear Medicine, Amsterdam UMC, Vrije Universiteit Amsterdam, Amsterdam 1007MB, The Netherlands; Centre for Medical Image Computing, Department of Computer Science, University College London, London WC1V 6LJ, UK; Centre for Medical Image Computing, Department of Medical Physics and Biomedical Engineering, University College London, London WC1V 6LJ, UK

**Keywords:** Alzheimer's disease, PART, data-driven subtyping, PET imaging, neuropathology

## Abstract

Amyloid-β is thought to facilitate the spread of tau throughout the neocortex in Alzheimer's disease, though how this occurs is not well understood. This is because of the spatial discordance between amyloid-β, which accumulates in the neocortex, and tau, which accumulates in the medial temporal lobe during ageing. There is evidence that in some cases amyloid-β-independent tau spreads beyond the medial temporal lobe where it may interact with neocortical amyloid-β. This suggests that there may be multiple distinct spatiotemporal subtypes of Alzheimer's-related protein aggregation, with potentially different demographic and genetic risk profiles. We investigated this hypothesis, applying data-driven disease progression subtyping models to post-mortem neuropathology and *in vivo* PET-based measures from two large observational studies: the Alzheimer's Disease Neuroimaging Initiative (ADNI) and the Religious Orders Study and Rush Memory and Aging Project (ROSMAP).

We consistently identified ‘amyloid-first’ and ‘tau-first’ subtypes using cross-sectional information from both studies. In the amyloid-first subtype, extensive neocortical amyloid-β precedes the spread of tau beyond the medial temporal lobe, while in the tau-first subtype, mild tau accumulates in medial temporal and neocortical areas prior to interacting with amyloid-β. As expected, we found a higher prevalence of the amyloid-first subtype among apolipoprotein E (APOE) ε4 allele carriers while the tau-first subtype was more common among APOE ε4 non-carriers. Within tau-first APOE ε4 carriers, we found an increased rate of amyloid-β accumulation (via longitudinal amyloid PET), suggesting that this rare group may belong within the Alzheimer's disease continuum. We also found that tau-first APOE ε4 carriers had several fewer years of education than other groups, suggesting a role for modifiable risk factors in facilitating amyloid-β-independent tau. Tau-first APOE ε4 non-carriers, in contrast, recapitulated many of the features of primary age-related tauopathy. The rate of longitudinal amyloid-β and tau accumulation (both measured via PET) within this group did not differ from normal ageing, supporting the distinction of primary age-related tauopathy from Alzheimer's disease. We also found reduced longitudinal subtype consistency within tau-first APOE ε4 non-carriers, suggesting additional heterogeneity within this group.

Our findings support the idea that amyloid-β and tau may begin as independent processes in spatially disconnected regions, with widespread neocortical tau resulting from the local interaction of amyloid-β and tau. The site of this interaction may be subtype-dependent: medial temporal lobe in amyloid-first, neocortex in tau-first. These insights into the dynamics of amyloid-β and tau may inform research and clinical trials that target these pathologies.

## Teaser

Aksman *et al.* model the spread of Alzheimer's disease pathologies using data-driven subtyping, identifying amyloid-first and tau-first subtypes. Amyloid-beta and tau may begin as independent processes in spatially disconnected regions, with local interaction of amyloid-beta and mild tau triggering widespread neocortical tau.

## Introduction

Alzheimer's disease is a progressive neurodegenerative disease that is characterized at the molecular level by the accumulation of two specific protein-based pathologies within the brain: amyloid plaques, composed of extracellular amyloid-β (Aβ) peptide, and intracellular neurofibrillary tangles (NFTs), composed of abnormally hyperphosphorylated tau protein. These pathologies combine to create a toxic environment that drives neurodegeneration via neuronal and synaptic loss, leading to cognitive impairment.^1^ While Aβ and tau have been recognized as the primary signature of Alzheimer’s disease, the causal relationship between these two pathologies is not fully understood. The prevailing view set forth by the amyloid cascade hypothesis is that the accumulation of Aβ peptides is the main causative event triggering the pathogenesis of Alzheimer’s disease, with tau-based NFTs, neurodegeneration and cognitive impairment following as a result.^2,3^

Importantly, the amyloid cascade hypothesis does not require that Aβ occurs first in all Alzheimer’s disease cases. Tau-based NFTs are well known to accumulate within the medial temporal lobe (MTL; includes entorhinal cortex, hippocampus and amygdala) in most individuals by their fifth or sixth decade in an age-related process that is independent of Aβ.^4,5^ Therefore, rather than occurring first, Aβ is thought to facilitate the spread of tau beyond the MTL.^6^ How this occurs is not well understood due to the spatial disconnection between Aβ accumulation, which usually begins in the parietal, cingulate and frontal regions in the neocortex,^7,8^ and age-related tau accumulation in the MTL.^9^ These pathologies may initiate independently and only interact when Aβ eventually spreads to the MTL. It is also possible that tau in the MTL somehow initiates neocortical Aβ,^10^ although a recent study in genetically identical twins supports the causal effect of Aβ on tau rather than the opposite.^11^ A third possibility is that tau spreads beyond the MTL in some cases^12^ and may interact locally with neocortical Aβ, which then amplifies tau. Taken together, these possibilities suggest that there may be two basic subtypes of pathology progression in Alzheimer’s disease: an ‘amyloid-first’ variant, in which widespread Aβ plaques precede neocortical NFTs, and a ‘tau-first’ variant, in which early neocortical NFTs precede widespread Aβ.

In this study we set out to investigate the existence of multiple spatiotemporal patterns of Aβ and tau progression using *in vivo* PET from the Alzheimer's Disease Neuroimaging Initiative (ADNI) and post-mortem neuropathological measures from the Religious Orders Study and Rush Memory and Aging Project studies (ROSMAP). We employed a data-driven paradigm to uncover subtypes of pathologic progression using the SuStaIn (Subtype and Stage Inference) algorithm.^13^ SuStaIn identifies groups of participants with common patterns of disease progression from multi-modal cross-sectional data. It has previously been used to establish the existence of multiple subtypes of both Aβ and tau spread.^8,14^ We consistently identified ‘amyloid-first’ and ‘tau-first’ progression patterns, each of which is marked by a distinct spatiotemporal pattern of Aβ and tau spreading. We then tested for differences in demographic and apolipoprotein E (APOE) ε4 status between these subtypes to better understand their relationship to Alzheimer’s disease and primary age-related tauopathy (PART^15^), the latter being characterized by age-related tau in the MTL in the absence of Aβ. Finally, using longitudinal Aβ and tau PET and cognition in ADNI, we investigated the longitudinal consistency of the PET-based subtyping model and tested for differences in the rates of Aβ and tau accumulation and cognitive decline between subtypes stratified by APOE ε4 status.

## Materials and methods

### ROSMAP dataset

We used post-mortem neuropathology data from the Religious Orders Study (ROS) and Rush Memory and Aging Project (MAP) studies, collectively referred to as ROSMAP, which we obtained from the Rush Alzheimer's Disease Center (RADC).^16^ Participants in these studies are cognitively normal (CN) older adults who agree to annual evaluations and organ donation as a condition of study entry. We used molecularly-specific immunohistochemistry-based measures of Aβ protein (per cent area of region occupied) and neuronal neurofibrillary tangles (associated with abnormally phosphorylated tau protein; cortical density per mm^2^ measured via AT8 staining) both measured in eight brain regions: hippocampus, entorhinal cortex, midfrontal cortex, inferior temporal cortex, angular gyrus, calcarine cortex, anterior cingulate cortex and superior frontal cortex. We also used demographic information (age at death, sex, education years), final (*in vivo*) clinical diagnosis of Alzheimer’s disease (NINCDS-ARDRA^17^), (post-mortem) neuropathological diagnosis of Alzheimer’s disease (NIA-Reagan Criteria^18^), Consortium to Establish a Registry for Alzheimer's Disease (CERAD) score (a semiquantitative measure of neuritic plaques^19^) and Braak stage (a semiquantitative measure of the distribution and severity of NFTs^20^).

### ADNI dataset

Data used in the preparation of this article were obtained from the ADNI database (adni.loni.usc.edu). The ADNI was launched in 2003 as a public-private partnership, led by Principal Investigator Michael W. Weiner, MD. The primary goal of ADNI has been to test whether serial MRI, PET, other biological markers and clinical and neuropsychological assessment can be combined to measure the progression of mild cognitive impairment (MCI) and early Alzheimer's disease (Alzheimer’s disease). For up-to-date information, see www.adni-info.org.

We downloaded and collated spreadsheets with imaging, demographic, cognitive and CSF measures from the ADNI IDA website. We downloaded regional amyloid PET (^18^F-AV-45, florbetapir) standardized update value ratios (SUVRs; UCBERKELEYAV45_8mm_02_17_23.csv) as well as both the standard regional tau PET (^18^F-AV-1451, flortaucipir) SUVRs (UCBERKELEYAV1451_8mm_02_17_23.csv) and partial volume corrected regional tau PET SUVRs (UCBERKELEYAV1451_PVC_8mm_02_17_23.csv). We also downloaded the ADNIMERGE table, containing demographic information (age, sex, years of education, number of APOE ε4 alleles), and diagnostic labels (CN/MCI/AD). We downloaded composite measures of memory (ADNI-MEM^21^) and executive function (ADNI-EF^22^) both available in UWNPSYCHSUM_12_13_21.csv. We download the following CSF spreadsheets: UPENNBIOMK9_04_19_17.csv (ADNI1/GO/2 Aβ_42_, pTau, tTau), UPENNBIOMK10_07_29_19.csv (ADNI3 Aβ_42_, Aβ_40_, pTau, tTau), UPENNBIOMK12_01_04_21.csv (additional ADNI3 Aβ_42_, Aβ_40_, pTau, tTau). The ADNI database was last accessed on 24 March 2023.

### Disease progression modelling

We used SuStaIn, a probabilistic machine learning method, to characterize the heterogeneity of Aβ and tau pathology progression in Alzheimer’s disease. SuStaIn infers multiple patterns of disease progression (i.e. subtypes) as well as individuals’ disease stages from cross-sectional data.^13^ The SuStaIn model as introduced by Young *et al*.^13^ uses a data likelihood based on how far a biomarker measurement deviates from normality, with an associated set of *z*-score based events for each biomarker. Note that in biomarkers where controls have very little abnormality, the resulting *z*-scores in patients can become large owing to the small amount of variance in the control population. This is indeed the case when modelling the progression of PET-based SUVRs, where the variability of the PET signal in the control group (e.g. Aβ load in cognitively normal APOE ε4 negative participants, representing normal ageing) can be quite small. We therefore followed the approach taken by Vogel *et al*.^14^ in our PET-based analysis, defining three events for each regional SUVR: *z* = 2, 5 and 10. These correspond roughly to mild, moderate and severe abnormality relative to the control group.

For our neuropathology-based analysis, we used an extension of SuStaIn (Ordinal SuStaIn^23^), that is adapted to handle severity scores from neuropathology rather than continuous values. This model was recently applied to model the progression of TDP-43 pathology using regional neuropathological severity score ratings, with each region assigned a score ranging from 0 (non-detectable) to 3 (severe).^24^ Because we did not have regional scores we estimated them by combining the quantitative, immunohistochemistry-based measures of pathology (Aβ and tau tangle severity in eight regions, described above) with CERAD scores for overall neuritic plaque burden (neuritic plaques are composed of insoluble Aβ) and Braak stages for overall NFT severity and spatial extent. We fit a kernel density estimation (KDE)-based probability distribution to the quantitative pathology measures associated with each CERAD or Braak score (or grouping of scores) and used a mixture-model based approach to assign a severity score probability to each individual in each region.

To do this we used the following procedure: for a set of regions i=1,…,I, participants j=1,…,J and unique severity scores k=1,…,K, we fit a KDE-based probability distribution p(x|score=k,region=i) to describe the probability of a pathology measure *x* in region *i* given score *k*, resulting in a mixture of *K* distributions per region. We performed the KDE mixture modelling in Python, using the gaussian_kde function in scikit-learn. In total we fit *I* × *K* distributions for all regions and severity scores. Following mixture modelling, we calculated $P(\mathrm{scor}e_{i,j,k})$, the probability of severity score *k* in region *i* for a given participant *j* with pathology measure $m_{ij}$ as:


(1) $$P(\mathrm{scor}e_{i,j,k})=\frac{\;p(m_{ij}\;|\;\mathrm{score}=k,\mathrm{region}=i)}{\sum_{k^{\prime }=1}^{K}\;P(m_{ij}\;|\;\mathrm{score}=k^{\prime },\mathrm{region}=i)}$$

where the numerator is the probability of observing the pathology measure under the probability distribution for score *k* in region *i*. The denominator assures that the severity score probabilities add up to one for each participant in each region.

We applied the above procedure to the set of Aβ measures and CERAD-based scores to generate a subjects × regions × scores matrix of severity score probabilities for regional Aβ severity. We applied the same procedure to the set of tau tangle measures and Braak-based scores to generate a second matrix of severity score probabilities for regional tau severity.

We used the pySuStaIn software package^25^ for both the PET-based *z*-score SuStaIn analysis and the neuropathology-based Ordinal SuStaIn analysis. In both cases we optimized the number of subtypes in an iterative manner using 10-fold cross-validation. Following previous SuStaIn-based studies,^13,14^ we evaluated the cross-validation information criterion (CVIC; described in Young *et al*.^13^). We chose the number of subtypes that consistently minimized the CVIC across both analyses.

### ROSMAP subtyping

The ROSMAP study is an ongoing observational study of older adults that have agreed to annual clinical evaluation and cognitive testing as well as brain donation after death. As of 2022, 3751 participants were enrolled, with 1853 deaths. There were a total of 1338 participants who had a complete set of Aβ and NFT measures for all eight available brain regions (hippocampus, entorhinal cortex, midfrontal cortex, inferior temporal, angular gyrus, calcarine cortex, anterior cingulate cortex, superior frontal cortex).

In order to run SuStaIn on these participants, we first took the square root of each measure to improve normality and then corrected each measure for the effect of normal ageing and normal demographic differences by training a region-specific regression model on a control population with the measure in question as the dependent variable and age at death, sex and education years as the independent variables. The control population consisted of 145 APOE ε4 negative (ε4−) cognitively normal participants (based on a summary diagnostic opinion regarding most likely clinical diagnosis at time of death) with a CERAD score of ‘no Alzheimer’s disease’, indicating very low or no neuritic plaques. We then residualized each region (true value minus predicted value from regression) and used these residualized measures in the mixture modelling procedure described above to estimate the regional score probability matrices for both Aβ and tau tangle pathologies.

For estimating regional Aβ score probabilities we combined the regional Aβ measures with the global CERAD score that was available for each participant. The CERAD score has four possible values: ‘no Alzheimer’s disease’, ‘possible Alzheimer’s disease’, ‘probable Alzheimer’s disease’ and ‘definite Alzheimer’s disease’. We used these directly to create four distributions for each region. For estimating regional tau tangle score probabilities we combined the regional NFT measures with each participant's Braak stage, which ranges from 0 (no NFTs), I and II (initial NFTs in entorhinal and early hippocampal regions), III and IV (worsening in previous regions and spread throughout temporal and cingulate regions) and V and VI (worsening in previous regions and spread to remaining cortex).^20^ In this case, to maintain consistency with the four Aβ severity scores, we grouped some Braak stages together, creating four tau severity scores. For the entorhinal and hippocampus regions the groups were: Braak 0/I/II (reflecting normal age-related tau in the MTL in those over 75^4^), Braak III/IV (mild), Braak V (moderate) and Braak VI (severe). For the other six regions, which become abnormal in later Braak stages (cingulate, calcarine, angular gyrus, inferior temporal, midfrontal, superior frontal) the groups were: Braak 0/I/II/III (none or minimal), Braak IV (mild), Braak V (moderate) and Braak VI (severe). We then followed the mixture modelling procedure with four severity scores for both Aβ and tau pathologies, generating a regional severity score probability matrix that were then combined and input to Ordinal SuStaIn.

### ADNI subtyping

We performed SuStaIn-based analyses using cross-sectional PET data from ADNI. We used 10 regional amyloid PET (AV-45) SUVRs and 12 tau PET (AV-1451) SUVRs, many of which were composites of several Freesurfer-based SUVRs (for complete details see Supplementary Table 1).^26,27^ We formed composite regions using volume-weighted averaging of two or more adjacent regions. We excluded the hippocampal tau PET SUVR as this region is suspected to be contaminated by off-target binding in the choroid plexus.^28^ We reference normalized all SUVRs as recommended for cross-sectional analysis: for amyloid PET we used a reference region made up of the whole cerebellum; for tau PET we used the inferior cerebellum in our main analysis and the inferior cerebellar grey matter for partial-volume corrected SUVRs for our supplementary analysis.^29,30^ For longitudinal analysis of Aβ and tau accumulation, we used the same reference region for tau PET and the recommended composite region (unweighted average of whole cerebellum, brainstem/pons and subcortical white matter) for amyloid PET.^29^

As in the ROSMAP analysis, we removed the associations with normal ageing and normal demographic factors by training a regression model for each biomarker's values against age, sex and education years in a control population of 49 cognitively normal participants who were APOE ε4−, global amyloid SUVR negative (whole cerebellum normalized summary SUVR < 1.11 cut-off^31,32^) and CSF Aβ negative (Aβ_42_/Aβ_40_ ratio > 0.06 cut-off^33^). We then regressed out the signal due to these factors from all markers. There were a total of 1645 participants with either amyloid PET or tau PET scans at a single visit, of which 796 had only amyloid PET and 327 had only tau PET. We built the main *z*-score SuStaIn model using the 502 participants who had complete concurrent amyloid and tau PET imaging. These were 47 cognitively normal, 406 with mild cognitive impairment (MCI) and 49 Alzheimer’s disease participants. To test the robustness of our main model, we used the same set of participants and trained an additional SuStaIn model with the same 10 amyloid PET SUVRs and partial volume corrected tau PET SUVRs for the same 12 composite regions.

We assessed the longitudinal consistency of the ADNI subtyping model using 170 participants who had concurrent amyloid and tau PET imaging at one or more follow-up visits. There were 210 follow-up samples in total: 22 at 1-year follow-up, 103 at 2-year follow-up, 13 at 3-year follow-up, 57 at 4-year follow-up, 10 at 5-year follow-up and five at 6-year follow-up. We created confusion matrices for subtype consistency within the APOE ε4− and ε4+ groups using the 103 participants with 2-year follow-up (58 ε4−, 45 ε4+).

### Statistical comparisons of early-stage groups

Following SuStaIn modelling, we tested for genetic and demographic differences between the stage-zero group (those assigned stage zero in either subtype, representing normal ageing) and those in the early stages of the amyloid-first and tau-first groups that we identified in both analyses. These early-stage groups included participants with abnormality in either Aβ or tau but not both at the same time to avoid the scenario in which SuStaIn cannot reliably disambiguate between subtypes based on a patients’ cross-sectional biomarker pattern. We stratified both the early amyloid-first and early tau-first groups by APOE ε4 carriage (ε4− versus ε4+) and tested for differences in Aβ and tau pathology across the five groups. For the neuropathology analysis, we tested for differences in Aβ in the angular gyrus and midfrontal regions (two of the earliest regions to show abnormality in our model) and for differences in tau tangles in the entorhinal cortex and hippocampus (two early Braak stage regions). For the PET analysis we tested for differences in Aβ pathology in the global amyloid SUVR and CSF Aβ_42_/Aβ_40_ ratio; for tau we tested for differences in the tau PET entorhinal regional SUVR. We also test for differences in CSF pTau. In each case we tested for differences across the five groups using three linear regressions, each time setting the regional measure as the dependent variable and sex, education years and group coding variables as the independent variables. In each case the first model included all groups, testing for differences relative to the stage zero reference group. The second model tested for differences within the two early amyloid-first groups (ε4+ versus ε4−). The third similarly tested for differences within the two early tau-first groups.

We then tested for demographic and genetic differences across these groups. We tested for differences in the proportion of early amyloid-first, early tau-first and stage-zero groups within APOE ε4− and ε4+ participants using a chi-squared test. As before, we tested for differences in age across the five groups using three linear regressions, each time setting age as the dependent variable and sex, education years and group coding variables as the independent variables. We tested for differences in sex using a set of three logistic regressions, each time setting sex as the dependent variable and age, education years and group coding variables as the independent variables. Finally, we tested for differences in education using a set of three linear regressions with education as the dependent variable and age, sex and group coding as the independent variables.

We investigated group differences in the rates of longitudinal Aβ and tau accumulation and cognitive decline using a set of linear mixed effects models (LMEs). All LME models were fitted using the *fitlme* function in Matlab (R2023a) with default parameters: using maximum likelihood with a full covariance matrix using Cholesky parameterization. For ROSMAP we modelled ante-mortem cognitive decline using all available longitudinal measures of global cognition, which is a composite measure of 19 cognitive tests that has been previously described by Bennett *et al*.^34^ For ADNI we modelled Aβ and tau accumulation using amyloid PET and tau PET measures and cognitive decline using composite memory score (ADNI-MEM) and composite executive function (ADNI-EF). For these models we used samples from all available visits (i.e. including visits that were both prospective and retrospective to the PET visit used in SuStaIn modelling) and used stage-zero (ε4−) participants as the reference group. For amyloid and tau PET we trained an LME model with fixed effects of baseline age, sex, education years, intracranial volume (ICV), time (years since baseline) and Time × Subtype interaction and individual-level random intercepts and random slopes with time. For the cognition models in ROSMAP and ADNI we used these same LME fixed and random effects, excluding ICV.

## Results

Demographics for the ROSMAP and ADNI cohorts used in our subtyping analyses are shown in Table 1. ROSMAP participants were older than ADNI participants (ROSMAP: 89.9 ± 6.4, ADNI: 75.2 ± 7.9 years; *P* < 10^−6^) while ADNI participants had more years of education (ROSMAP: 15.9 ± 3.6, ADNI: 16.4 ± 2.6 years; *P* = 0.005). ROSMAP had a higher proportion of females (ROSMAP: 69%, ADNI: 50%; *P* < 10^−6^) while ADNI had a higher proportion of APOE ε4 carriers [ROSMAP: 76%/22%/2% (0/1/2 alleles), ADNI: 65%/28%/7% (0/1/2 alleles); *P* < 10^−6^].

**Table 1:** Characterization and comparison of subtyping cohorts

	ROSMAP	ADNI	
*n*	1338	502	
Age, mean ± SD [min, max]	89.9 ± 6.4 [65.9, 108.3]	75.2 ± 7.9 [55.3, 93.8]	<1 × 10^−6^***
Education years, mean ± SD [min, max]	15.9 ± 3.6 [3.0, 30.0]	16.4 ± 2.6 [8.0, 20.0]	0.005**
Females, %	69%	50%	<1 × 10^−6^***
APOE ε4 alleles (% 0,1,2)	76%, 22%, 2%	65%, 28%, 7%	<1 × 10^−6^***

APOE ε4 was available for all ROSMAP participants and 470 ADNI participants. We compared age and education years via one-way ANOVAs and sex and APOE ε4 carriage via chi-squared tests. SD = standard deviation. *P*-values of these tests are reported in right-hand column.

*P < 0.05, **P < 0.01, ***P < 0.001.

### Amyloid-first and tau-first subtypes

Supplementary Fig. 1 depicts the mixture models that were fit for the ROSMAP analysis. We used these models to generate the regional severity score probability matrices, which were combined and input to Ordinal SuStaIn. Supplementary Fig. 2 depicts the distribution of *z*-scores for cognitively normal, MCI and Alzheimer’s disease participants’ SUVRs in ADNI, showing that cognitively normal and MCI participants’ *z*-scores are generally small (with higher variability of scores within the MCI group) and Alzheimer’s disease participants’ *z*-scores are substantially higher, as expected. We used these *z*-scores as input to *z*-score SuStaIn.

We estimated the number of subtypes that best explain the progression of Aβ and tau pathology in both datasets. To do this we built separate SuStaIn models for each dataset, allowing SuStaIn to infer one, two or three-subtype models in each case and we chose the most parsimonious models across both datasets. Supplementary Fig. 3 depicts the CVIC (lower is better) for both datasets. We chose the two-subtype models for all subsequent analyses as there was a consistent improvement over a one-subtype model in both analyses.

**Figure 1:**
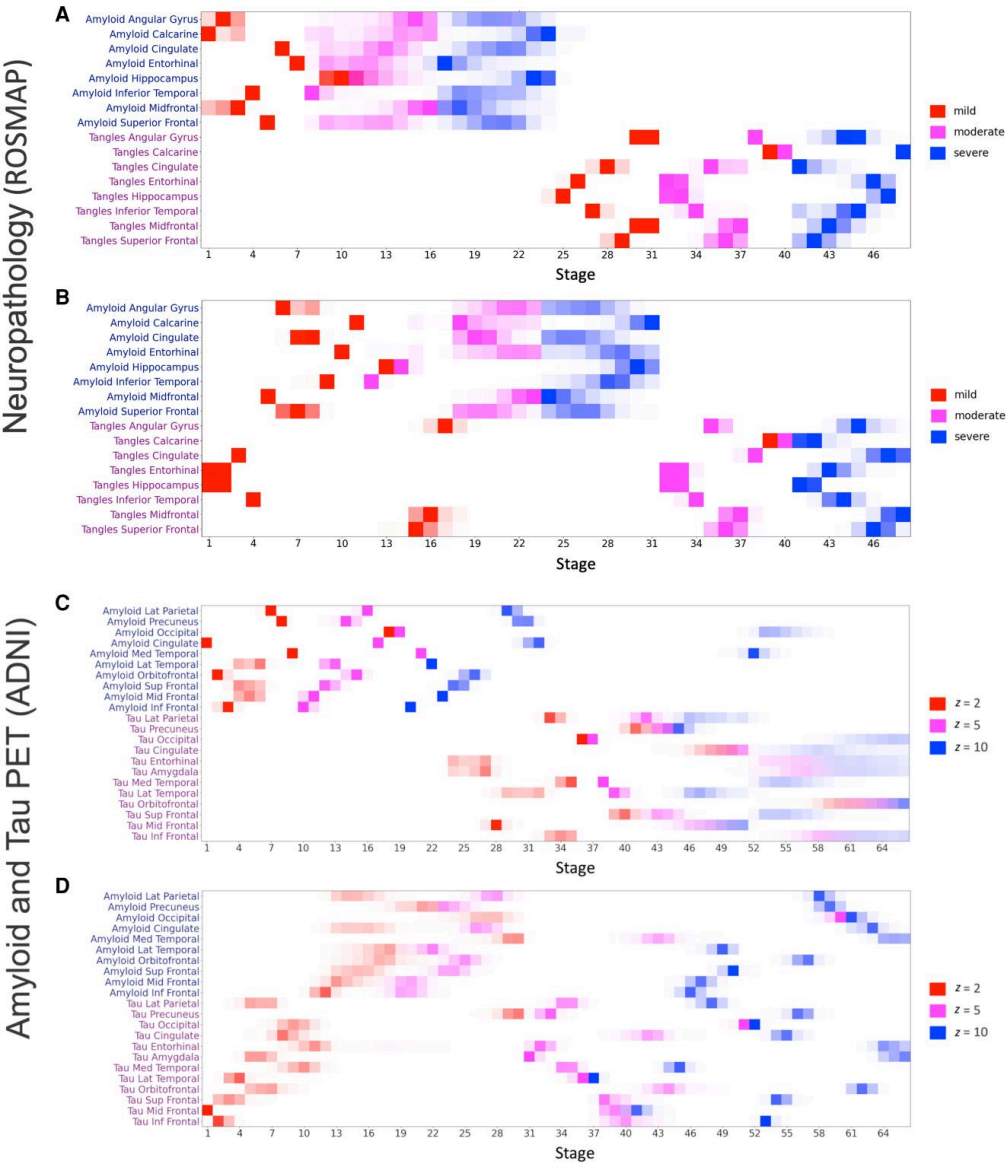
**Positional variance diagrams (PVDs) for two-subtype SuStaIn models.** Each panel represents a subtype, i.e. a unique pattern of disease progression from early to late stage disease. (**A** and **B**) PVDs for two-subtype model trained on trained on ROSMAP's neuropathology data. **A** is the ‘amyloid-first’ subtype, **B** is the ‘tau-first’ subtype. (**C** and **D**) PVDs for two-subtype model trained on ADNI's amyloid and tau PET SUVR data. **C** is the ‘amyloid-first’ subtype, **D** is the ‘tau-first’ subtype. Each coloured box represents the degree of certainty that a given regional marker (*y*-axis) has reached a given severity stage at a given SuStaIn stage (*x*-axis).

Based on the two-subtype models we chose, Fig. 1 depicts the positional variance diagrams (PVDs) representing the progression patterns estimated by SuStaIn. Each PVD visualizes event sequence uncertainties as a matrix where each row presents a set of three histograms, one per event, that are represented by coloured boxes. In both analyses, each region has three stages of increasing abnormality relative to a control group that is expected to be at minimal risk of Alzheimer’s disease (in both cases: amyloid-negative, APOE ε4−, cognitively normal participants).

Across both analyses we consistently found an ‘amyloid-first’ and a ‘tau-first’ subtype. In the neuropathology analysis, the ‘amyloid-first’ subtype is characterized by the initial spread of Aβ plaques throughout the cortex and MTL (here represented by the hippocampus and entorhinal cortex). Following severe Aβ plaques in all regions, mild tau tangle pathology in the hippocampus and entorhinal cortex (exceeding Braak I/II severity expected in normal ageing) spreads to the inferior temporal lobe and throughout the neocortex (Fig. 1A). The latter stages of this subtype are marked by increasing tau tangle pathology, which progresses from mild to moderate to severe. The ‘tau-first’ subtype is characterized initially by mild tau tangle pathology in the entorhinal cortex, hippocampus, inferior temporal lobe and cingulate. Mild tau in these regions is followed by the spread of Aβ plaques throughout the brain, with subsequent increase in tau tangle pathology throughout the MTL and neocortex (Fig. 1B).

In the PET-based analysis the ‘amyloid-first’ subtype is initially marked by the spread of Aβ that progresses to a severity that is at least 5 standard deviations (SD) from normality in all regions. Following this, mild tau accumulates in the entorhinal cortex and amygdala (beyond what is expected in normal ageing, with hippocampus excluded in this analysis) and spreads throughout the cortex, with increased severity of both Aβ and tau pathologies (Fig. 1C). The ‘tau-first’ subtype is marked by mild tau abnormality in all regions (*z*-scores of 2 in frontal, temporal, parietal, occipital and cingulate regions), followed by the spread of Aβ throughout the cortex (up to a *z*-score of 5 in most regions) with subsequent increased tau severity in all regions (Fig. 1D).

We built several additional SuStaIn-based subtyping models to test the robustness of our findings. The first two were based on the CVIC figure in Supplementary Fig. 3, which showed a slightly lower CVIC for a three-subtype model rather than a two-subtype in the case of the PET-based analysis. For the sake of completeness, we present the three-subtype model for both datasets in Supplementary Figs 5 and 6. Increasing to three subtypes consistently creates an additional ‘tau-first’ subtype in which tau in the MTL (entorhinal cortex and hippocampus in the neuropathology model, entorhinal cortex and amygdala in the PET-based model) precedes Aβ. The third model substituted partial volume corrected tau PET SUVRs in place of standard SUVRs in the PET-based model. Supplementary Fig. 7 presents this model, which is very similar to the main PET-based model presented in Fig. 1C and D.

### Amyloid and tau differences among early-stage groups

For the neuropathology model we defined the early amyloid-first group as those with moderately abnormal Aβ and no abnormal tau (stages 1 to 16 in Fig. 1A, *n* = 168; APOE ε4−: 135, APOE ε4+: 33) and the early tau-first group as those with mild tau and no abnormal Aβ (stages 1 to 4 in Fig. 1B, *n* = 151; ε4−: 142, ε4+: 9). The stage zero group was composed of *n* = 106 participants in this case. For the PET-based model the early amyloid-first group was defined as those with *z* = 2 level abnormality in most regional amyloid PET SUVRs and no abnormal tau (stages 1 to 9 in Fig. 1C, *n* = 87; APOE ε4−: 50, APOE ε+: 37) and the early tau-first group as those with *z* = 2 level abnormality in nearly all tau PET SUVRS and no abnormal Aβ (stages 1 to 9 in Fig. 1D, *n* = 72; APOE ε4−: 62, APOE ε+: 10). The stage zero group was composed of *n* = 120 participants in this case.

**Figure 2:**
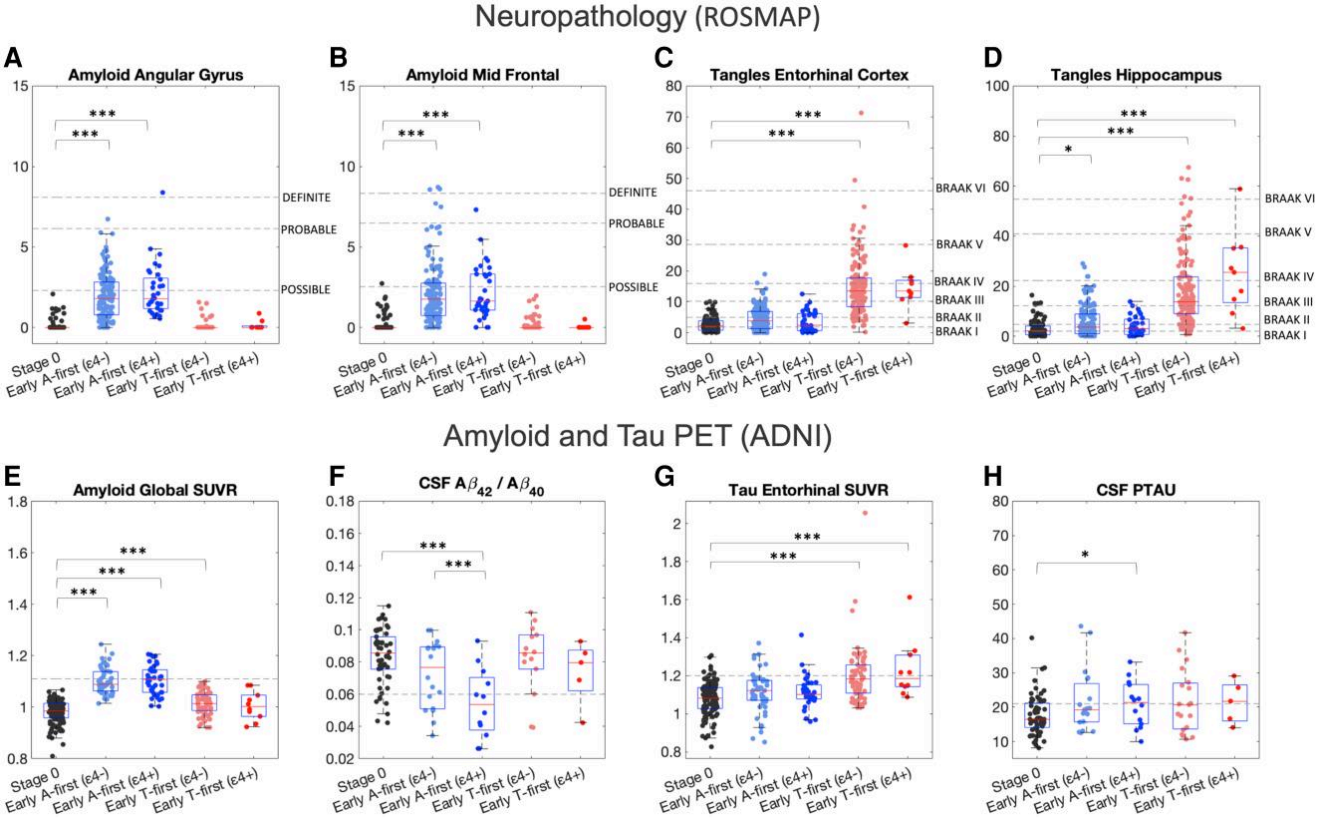
**Differences in Aβ and tau measures across early-stage groups.** *Top*: Pathology measures across early-stage groups in the neuropathology analysis. (**A** and **B**) Raw Aβ plaque measures (percentage of region) in the angular gyrus and midfrontal regions, showing the expected increase in Aβ plaques in the two early amyloid-first groups (APOE ε4−, ε4+) with reference lines based on average values of those diagnosed as possible, probable and definite Alzheimer’s disease based on CERAD scoring of neuritic plaques. (**C** and **D**) Raw tangle density measures (per mm^2^) in the entorhinal and hippocampal regions, showing the expected increase in the two early tau-first groups with reference lines based on average values of those assigned Braak I–VI stages. *Bottom*: Biomarker measures across early-stage groups in the PET-based analysis. (**E**) Amyloid PET global SUVR, showing expected increase in both early amyloid-first groups and a small increase in early tau-first group (ε4−). Reference line: amyloid PET positivity threshold of 1.11 or greater. (**F**) CSF Aβ_42_/Aβ_40_ ratio, showing decreased ratio (increased Aβ deposition) in early amyloid-first (ε4+) group relative to both early amyloid-first (ε4−) and stage zero groups. Reference line: CSF Aβ_42_/Aβ_40_ ratio positivity threshold of 0.06 or less. (**G**) Tau PET entorhinal region SUVR, showing expected increase in tau pathology in both early tau-first groups. Reference line: regional positivity threshold of 1.2 or greater. (**H**) CSF pTau, showing small increase in early amyloid-first (ε4+). Reference line: positivity threshold of 21 or greater. SUVR = standardized update value ratio.

For the neuropathology model we found the expected increase in Aβ in the angular gyrus and mid-frontal regions within both early amyloid-first groups relative to the stage zero group (ε4−: angular gyrus *t* = 14.4, *P* < 10^−6^, midfrontal *t* = 12.3, *P* < 10^−6^; ε4+ angular gyrus *t* = 11.6, *P* < 10^−6^; midfrontal t = 8.4, *P* < 10^−6^) (Fig. 2A and B). Similarly, we found increased tau tangles in the entorhinal cortex and hippocampus in both early tau-first groups relative to the stage zero group (ε4−: entorhinal cortex *t* = 15.1, *P* < 10^−6^, hippocampus *t* = 13.1, *P* < 10^−6^; ε4+: entorhinal cortex *t* = 5.1, *P* < 10^−6^, hippocampus *t* = 6.4, *P* < 10^−6^) (Fig. 2C and D). We also found a small increase in tau tangles in the hippocampus in the early amyloid-first group (ε4−) relative to the stage-zero group (*t* = 2.1, *P* = 0.04) (Fig. 2D).

For the PET-based model we found the expected increase in global amyloid PET SUVR within both early amyloid-first groups relative to the stage zero group (ε4−: *t* = 16.1, *P* < 10^−6^, ε4+: 14.5, *P* < 10^−6^) (Fig. 2E). We also found a small increase in global amyloid PET SUVR in the early tau-first group (ε4−) versus the stage-zero group (*t* = 5.3, *P* < 10^−6^) (Fig. 2E). We found decreased CSF Aβ_42_/Aβ_40_ ratio (indicative of increased Aβ deposition) in the early amyloid-first (ε4+) group relative to both the early amyloid-first (ε4−) group and the stage-zero group (ε4+ versus stage-zero: *t* = −5.0, *P* < 10^−6^; ε4+ versus ε4−: *t* = −3.0, *P* = 0.006) (Fig. 2F). We also found the expected increase in entorhinal region tau PET SUVR signal in both early tau-first groups relative to the stage-zero group (ε4−: *t* = 7.2, *P* < 10^−6^; ε4+: *t* = 4.8, *P* = 2.8 × 10^−6^) (Fig. 2G). Finally, we found a small increase in CSF pTau in the early amyloid-first (ε4+) group relative to the stage-zero group (*t* = 2.0, *P* = 0.04) (Fig. 2H).

### Higher proportion of early amyloid-first group within APOE ε4 carriers

**Figure 3:**
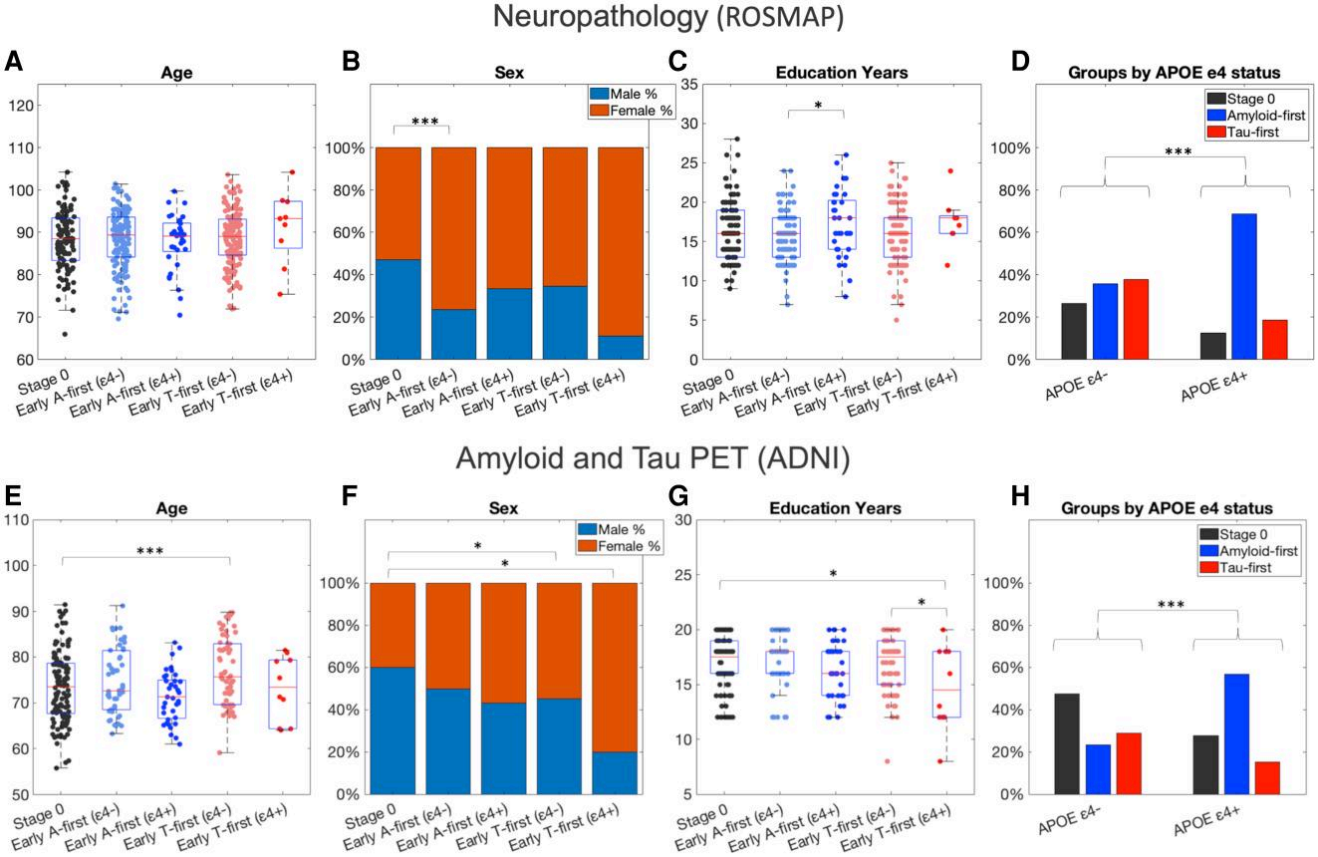
**Demographic measures across early stage groups along with a comparison of proportion of each group within APOE ε4+ and ε4− participants**. *Top*: ROSMAP neuropathology analysis, showing (**A**) no differences in age between groups; (**B**) early amyloid-first (ε4+) group has a higher proportion of females than the stage zero group; (**C**) small increase in years of education in early amyloid-first (ε4+) versus early amyloid-first (ε4−) group; and (**D**) higher prevalence of early amyloid-first group within ε4+ participants. *Bottom*: ADNI PET-based analysis, showing (**E**) small increase in age in early tau-first (ε4+) group relative to stage zero group; (**F**) higher proportion of females in early tau-first groups relative to stage zero group; (**G**) fewer years of education in the early tau-first (ε4+) group versus both early tau-first (ε4−) and stage zero groups; and (**H**) as in neuropathology analysis, a higher prevalence of early amyloid-first group within ε4+ participants.

We consistently found that APOE ε4+ participants were more likely to belong to the early amyloid-first group than ε4− participants (neuropathology model: 69% of ε4+ in early amyloid-first group versus 36% of ε4− participants, chi-squared = 19.3, *P* = 6.3 × 10^−5^; PET-based model: 57% ε4+ versus 23% ε4−, chi-squared = 26.2, *P* = 2.0 × 10^−6^) (Fig. 3D and H). Within the neuropathology model we also found a higher proportion of females in the early amyloid-first (ε4−) group than in the stage zero group (early amyloid-first, ε4− group: 76% female, stage zero group: 52% female, odds ratio: 2.8, *P* = 3.4 × 10^−4^) (Fig. 3B) and a small increase in years of education in the early amyloid-first (ε4+) group compared to the early amyloid-first (ε4−) group (mean ± SD: 17.4 ± 4.3 years versus 16.4 ± 3.8 years; *t* = 2.5, *P* = 0.01) (Fig. 3C). Within the PET-based model we found those in the early tau-first (ε4−) group were slightly older and more likely to be female than those in the stage-zero group (age: 76.9 ± 7.4 years versus 73.4 ± 7.7 years, *t* = 3.6, *P* = 4.1 × 10^−4^; Fig. 3E; sex: 55% female versus 40% female, odds ratio: 2.4, *P* = 0.01) (Fig. 3E and F). Those in the early tau-first (ε4+) group were also more likely to be female compared to those in the stage zero group (80% versus 40%, odds ratio: 5.4, *P* = 0.04) (Fig. 3F). In addition, those in the early tau-first (ε4+) group had fewer years of education than both the early tau-first (ε4−) group and the stage zero group (ε4+: 14.7 ± 3.8 years, ε4−: 16.9 ± 2.5 years, stage zero: 16.8 ± 2.6 years; ε4+ versus ε4−: *t* = −2.1, *P* = 0.04; ε4+ versus stage zero: *t* = −2.2, *P* = 0.03).

### Longitudinal consistency of tau-first subtype depends on APOE ε4 status

**Figure 4:**
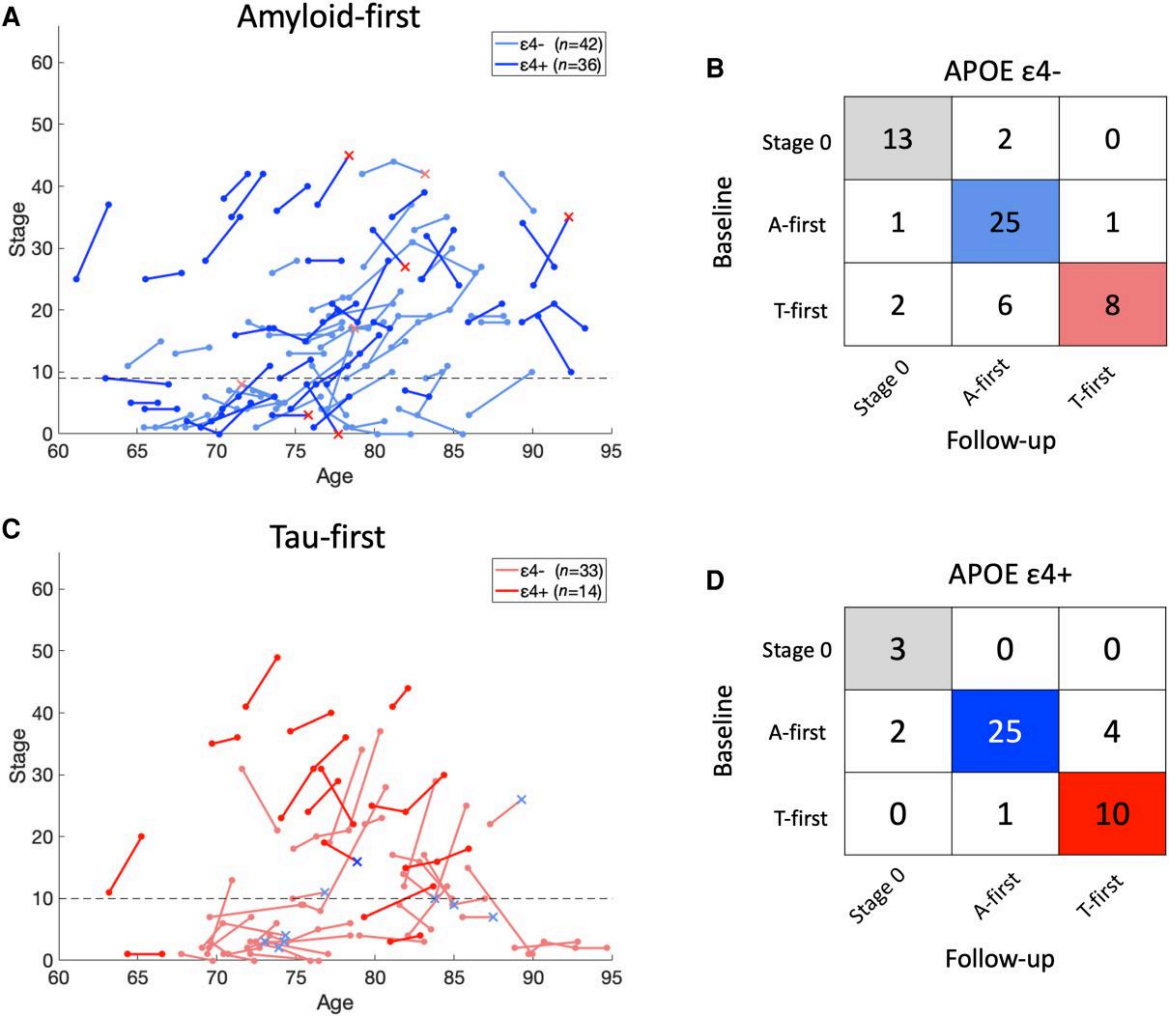
**Longitudinal consistency of PET-based model.** On the *left* are spaghetti plots of participants with either amyloid-first (**A**; *n* = 78) or tau-first (**C**; *n* = 47) as their estimated baseline subtype, stratified by APOE ε4 status within each figure. Each participant's longitudinal stage progression is depicted as a connected line, with opposite colours and ‘x’ markers used for points where the follow-up subtype is not consistent with the baseline subtype. The dashed lines represent the early-stage cut-off for each subtype (amyloid-first: stage 9, tau-first: stage 10). On the right are confusion matrices built by comparing each participant's estimated baseline subtype to their estimated 2-year follow-up subtype, stratified by APOE ε4 status (**B**: *n* = 58 ε4−, **D**: *n* = 45 ε4+).

We visualized the longitudinal consistency of the PET-based model with spaghetti plots of all available follow-up samples, showing the expected increase in stage over time in the majority of participants (Fig. 4A and C). Within the 103 participants with 2-year follow-up, we found no difference in the annual rate of stage increase between subtypes in either ε4− or ε4+ participants (ε4−, *n* = 58: amyloid-first: 0.6 ± 2.1 stages/year, tau-first: 0.9 ± 3.9 stages/year, one-way ANOVA *P* = 0.75; ε4+, *n* = 45: amyloid-first: 0.8 ± 2.7 stages/year, tau-first: 0.9 ± 2.7 stages/year, *P* = 0.87). Within ε4− participants, the tau-first group had a lower 2-year longitudinal consistency than the amyloid-first group (amyloid-first: 25 of 27, 93%; tau-first: 8 of 16, 50%; Fig. 4B; Fisher's exact test *P* = 0.003). There was no such difference within ε4+ participants, where the 2-year longitudinal consistency was high for both subtypes (amyloid-first: 25 of 31, 81%; tau-first: 10 of 11, 91%; Fig. 4D, *P* = 0.65).

### Amyloid accumulation within tau-first subtype depends on APOE ε4 status

**Figure 5:**
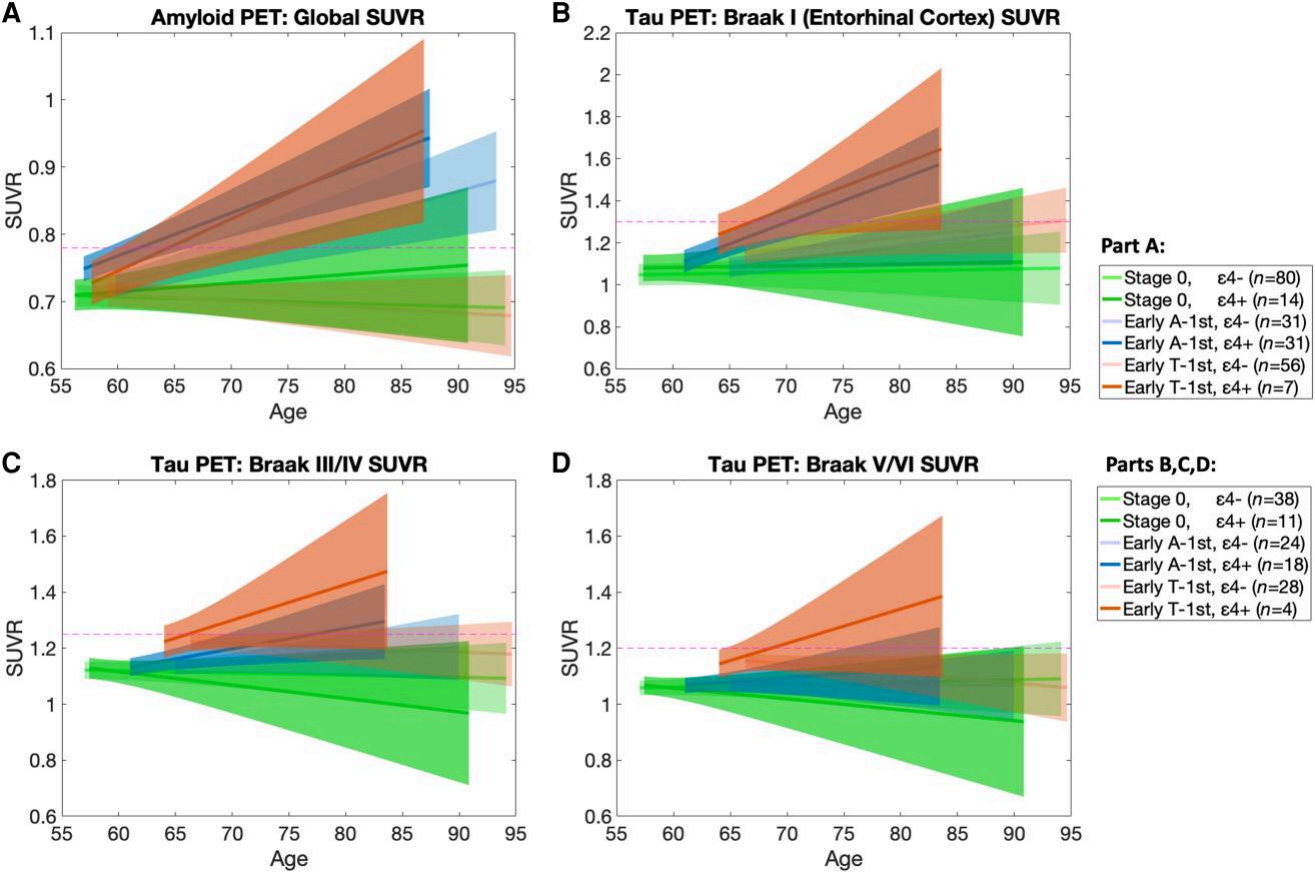
**Longitudinal amyloid and tau PET SUVR trajectories for early-stage groups in PET-based model based on linear mixed effects models**. (**A**) Amyloid PET-based global standardized update value ratio (SUVR) trajectories using composite reference region that is recommended for longitudinal analysis, with an abnormality cut-off of 0.78 as reference line. (**B**–**D**) Tau PET-based Braak composite SUVR trajectories with empirically chosen abnormality cut-offs based on distributions presented in Supplementary Table 8 (1.3 for Braak I in **B**, 1.25 for Braak III/IV in **C**, 1.2 for Braak V/VI in **D**).

Figure 5A depicts longitudinal trajectories of Aβ accumulation across early-stage groups from the PET-based model. We found increased intercepts and rates of amyloid accumulation within both early amyloid-first groups relative to stage zero (ε4−: intercept *t* = 2.6, *P* = 8.74 × 10^−3^, group × time interaction: *t* = 3.9, *P* = 8.79 × 10^−5^; ε4+: intercept *t* = 5.7, *P* < 10^−6^, group × time interaction: *t* = 5.0, *P* < 10^−6^) (Supplementary Table 3A). While these findings were expected for these groups, we also found an increased intercept and rate of Aβ accumulation within the early tau-first (ε4+) group, though longitudinal information was limited for this group (*n* = 7; intercept *t* = 2.0, *P* = 0.04, group × time interaction: *t* = 3.4, *P* = 6.26 × 10^−4^) (Supplementary Table 3A). We found no corresponding increase in Aβ accumulation within the early tau-first (ε4−) group relative to stage zero (*n* = 31; Supplementary Table 3A).

Figure 5B–D depict longitudinal trajectories of tau accumulation within composite Braak regions. We found increased intercepts for both early tau-first groups within all three composite regions relative to the stage zero group (Braak I, ε4−: *t* = 2.7, *P* = 6.65 × 10^−3^, ε4+: *t* = 3.6, *P* = 3.44 × 10^−4^; Braak III/IV, ε4−: *t* = 8.0, *P* < 10^−6^, ε4+: *t* = 3.8, *P* = 1.50 × 10^−4^; Braak V/VI, ε4−: *t* = 10.4, *P* < 10^−6^, ε4+: *t* = 4.2, *P* = 3.18 × 10^−5^) (Supplementary Table 3B–D). We found no corresponding differences in the rates of tau accumulation within these regions in either early tau-first group, suggesting that these groups have a high baseline level of tau but do not accumulate tau any faster than normal.

The early amyloid-first (ε4+) group was the only group in which we found increased tau accumulation, within both the Braak I and Braak III/IV composite regions (Braak I: *t* = 4.1, *P* = 4.71 × 10^−5^; Braak III/IV: *t* = 2.3, *P* = 0.02) (Supplementary Table 3B and C). We found no corresponding differences in intercepts in these regions within this group, suggesting that this group begins accumulating tau at an abnormally fast rate following widespread Aβ. We also found a small increase in intercept in the amyloid-first (ε4−) group within the Braak V/VI region, but no corresponding increase in the rate of tau accumulation (Braak V/VI: *t* = 2.4, *P* = 0.02), which may be due to additional heterogeneity within the ε4− group that is not well explained by our two-subtype model.

Finally, we found no differences in the rates of ante-mortem global cognitive decline in any of the four early-stage groups relative to the stage zero group within our neuropathology dataset (Supplementary Table 4A and Supplementary Fig. 4A). Within ADNI (PET-based model) we similarly found no increased rates of memory or executive function decline across early-stage groups and only a small difference in executive function intercept in the early tau-first (ε4+) group relative to the stage-zero group (*t* = −2.1, *P* = 0.04) (Supplementary Table 4B and C and Supplementary Fig. 4B and C).

## Discussion

While Aβ and tau have long been established as the main pathological hallmarks of Alzheimer’s disease, the heterogeneity within the spatiotemporal progression of these pathologies has yet to be fully understood. Here we performed data-driven modelling on two large cohorts with complementary *in vivo* and post-mortem measures, consistently finding ‘amyloid-first’ and ‘tau-first’ subtypes across both studies (Fig. 1). In the ‘amyloid-first’ subtype, widespread Aβ throughout the neocortex and the MTL precedes neocortical tau. This supports the idea that a spatially and temporally localized interaction between Aβ and age-related tau in the MTL (Fig. 2C and D) may trigger the spread of tau beyond the MTL (Fig. 1A and C). The ‘tau-first’ subtype is marked by mild tau in the MTL and, in some cases, the neocortex (cingulate and inferior temporal lobe in the neuropathology-based model; all available cortical regions in PET-based model) preceding Aβ (Fig. 1B and D). This finding supports *in vivo* tau PET studies,^12,35,36^ neuropathology studies^37,38^ and a recent combined study,^39^ which have found that mild tau may spread beyond the MTL in the presence of little or no Aβ. Our findings suggest that, in both subtypes, substantial neocortical tau accumulation may only occur after local interactions with Aβ. Importantly, the site of these interactions may differ between subtypes: in the amyloid-first subtype it occurs in the MTL (around stage 25 in Fig. 1A and stage 23 in Fig. 1C) while in the tau-first subtype it may occur in one or more neocortical regions where early Aβ deposition takes place (frontal, parietal or cingulate regions; around stage 5 in Fig. 1B and around stage 13 in Fig. 1D).

Beyond identifying these subtypes across complementary studies, our most important findings relate to their interaction with APOE ε4 status. Comparing the early stages of both subtypes, we found a higher prevalence of the amyloid-first subtype among ε4 carriers and, conversely, a higher prevalence of the tau-first subtype among ε4 non-carriers (Fig. 3D and H). Within the amyloid-first subtype, we found that ε4 carriers had greater Aβ deposition than ε4 non-carriers (lower Aβ_42_/Aβ_40_ ratio, Fig. 2F). These findings are consistent with studies showing that APOE ε4 carriage is associated with increased Aβ deposition^40,41^ and a higher lifetime risk of developing Alzheimer’s disease dementia.^42,43^ Although we expected earlier Aβ deposition in ε4 carriers versus non-carriers,^44^ we did not observe this in the PET-based analysis (Fig. 3E). This may be because our criteria for defining the early amyloid-first groups was based on most regions having the mildest Aβ accumulation (*z*-scores of 2 in most amyloid SUVRs), which may have been reached many years before our study baseline (average age of participants in PET-based analysis was 75.2 ± 7.9 years; Table 1).^44^ Consistent with this interpretation, we found both a higher baseline level and rate of Aβ accumulation in the early amyloid-first ε4 carriers compared non-carriers (Supplementary Table 3A).

Within the tau-first subtype we found an increased rate of Aβ accumulation in ε4 carriers compared to our normal ageing reference group, suggesting that this rare group may belong within the Alzheimer’s disease continuum (9 of 1338 participants in neuropathology dataset: 0.7%; 10 of 502 participants in ADNI: 2%; similarly infrequent in previous studies^45,46^). Interestingly, we found that those in the early tau-first (ε4+) group had several fewer years of education than other early-stage groups (Fig. 3G). This suggests a role for modifiable risk factors, such as reduced years of education^47^ or possibly head injury,^48^ in facilitating Aβ-independent neocortical tau in those who would normally develop neocortical tau only after substantial Aβ accumulation.

The tau-first (ε4−) group recapitulates key features of PART, which is characterized by tau pathology in the absence of Aβ plaques.^15,49,50^ The rate of both Aβ and tau accumulation within this group did not differ from normal ageing despite increased baseline tau in both the MTL and neocortex (Fig. 5 and Supplementary Table 3). Together with the older average age of this group (Fig. 3E), this suggests a very slow process of tau accumulation over a number of years, beginning in middle age or even earlier.^4,51^ This makes it hard to determine the exact sequence of progression of amyloid-independent tau. While our findings suggests that PART may be more closely related to normal ageing than Alzheimer’s disease, our conclusions are tempered by our finding that the tau-first (ε4−) group had substantially lower longitudinal subtype consistency than other groups (Fig. 4). The explanation for this may be that some of those who start out with mild tau in the MTL and/or neocortex and no Aβ subsequently develop low levels of Aβ, leading our model to misclassify their follow-up measures. These findings raise the question of whether: (i) the tau-first (ε4−) group represents PART, which is itself naturally heterogeneous and includes the roughly 30% of ε4 non-carriers who develop low levels of Aβ by their eight decade^44^; or (ii) those with PART are somehow protected from Aβ and therefore the tau-first (ε4−) group includes both PART and those on a slow trajectory of Aβ accumulation. These observations, which support several recent studies,^52,39^ motivate the need to identify and track early tau-first, ε4 non-carriers to better understand the heterogeneity within this group.

**Figure 6:**
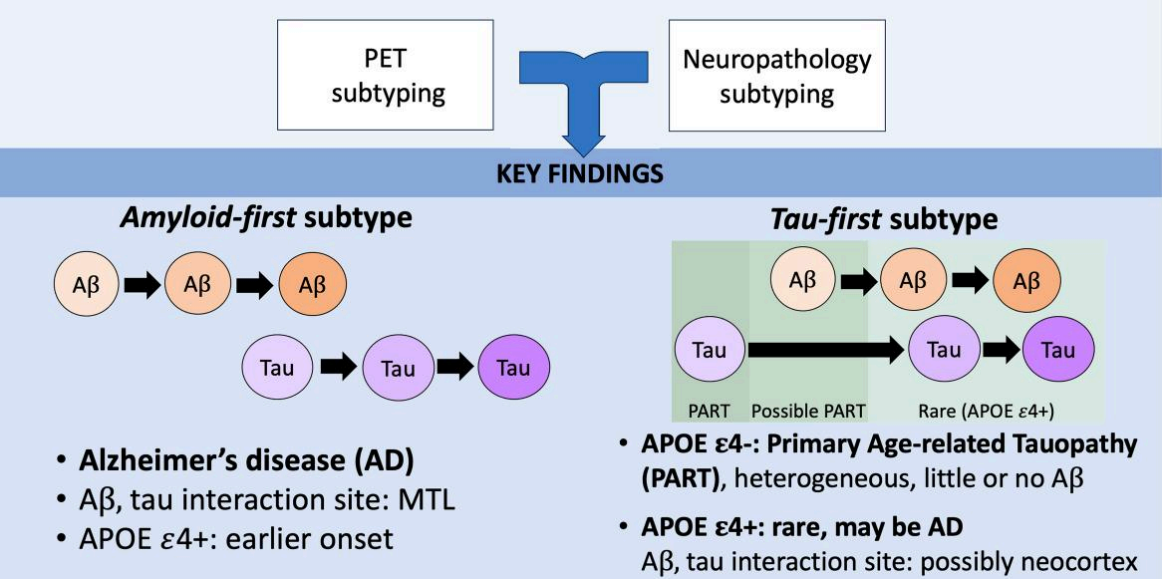
**Proposed model of Aβ and tau spread based on our findings**. We consistently identified amyloid-first and tau-first subtypes based on PET and neuropathology measures. The amyloid-first subtype represents the typical course of AD progression in which amyloid-β (Aβ) initially spreads throughout the cortex, represented by the lightest orange circle in the figure. Moderate-to-severe Aβ, represented by the darker orange circles, eventually interacts with age-related tau within the MTL, setting off the spread of tau throughout the neocortex. Mild, moderate and severe tau are represented by the purple circles. In APOE ε4 carriers this process may happen at an earlier age due to earlier Aβ accumulation. The tau-first subtype is marked by the initial accumulation of mild tau in the MTL and/or neocortex. Tau-first APOE ε4 non-carriers recapitulate the features of PART and are either partially or completely protected from Aβ accumulation. Tau-first APOE ε4 carriers, which are rare, may belong within the AD continuum based on their increased rate of Aβ accumulation. Within this group the site of interaction between moderate-to-severe Aβ and mild tau may take place in either the neocortex or MTL, which then accelerates the spread of neocortical tau.

Our tau PET sample is insufficient to validate the four PET-based tau subtypes found by Vogel *et al*.^14^ based on a larger sample size of 1143 tau PET images. However, our findings may help to explain some of the tau heterogeneity in those who are Aβ positive.^53^ Notably, the limbic-predominant subtype, which is characterized by Braak-like tau progression, has been found to have a higher proportion of APOE ε4 carriers. This is consistent with ε4 carriers having an earlier age of Aβ accumulation^44^ and therefore we expect the amyloid-first (ε4+) group to be primarily composed of the limbic-predominant subtype. Interestingly, increased Aβ deposition within amyloid-first ε4 carriers relative to non-carriers (Fig. 2F) may be related to the increased severity of MTL tangles within the limbic-predominant subtype. Correspondingly, we expect the amyloid-first (ε4−) group to be mostly composed of the other known tau subtypes (MTL-sparing, posterior and lateral temporal^14^). Importantly, once Aβ takes off we expect that it accelerates the spread of tau in all scenarios, consistent with the Aβ cascade hypothesis. The resulting picture is one of a slow tau accumulation process that is accelerated following local interaction with Aβ. The age and location at which this interaction takes place may depend on both genetic and modifiable risk factors of Aβ accumulation.^51^ The spatial variability in how tau spreads may also depend on these factors plus individual-level and population-level factors.^54^ Within this model, APOE ε4 non-carriers with PART are either partially or completely protected from Aβ while a small number of APOE ε4 carriers will develop abnormal tau prior to Aβ, possibly due to modifiable risk factors. While this model, summarized in Fig. 6, is probably an oversimplification it may be useful for future studies.

Our study has several important limitations. The first relates to the current lack of sufficiently long follow-up measures in the ADNI3 data, which may be remedied in ADNI4.^55^ This limited our validation of subtype consistency, which is important when using the SuStaIn algorithm to infer longitudinal progression patterns from cross-sectional observations. This is because there is a theoretical possibility of inferring a progression pattern from a set of unrelated disease states. A related methodological limitation is the crossing problem, in which two or more subtypes have middle stages that look identical (e.g. an individual with mild tau plus Aβ may belong to either subtype). In our study we accounted for this problem by focusing on the early stages of each subtype. A version of SuStaIn that is explicitly longitudinally consistent, so that each individual is guaranteed to be assigned to the same subtype over multiple observations, is being developed to address these limitations.^56^ There are also limitations related to comparing neuropathological measures from ROSMAP with *in vivo* measures from ADNI. The eight regional measures of Aβ and tau tangles measures used in neuropathological model were not anatomically consistent with the PET-based regional SUVRs, limiting our comparison of spatial progression patterns. This is especially evident in the tau-first subtype, where the lack of neuropathological measures in the precuneus, inferior frontal and orbitofrontal regions limited our ability to validate the PET-based finding that these may be among the earliest sites of tau and Aβ interaction (rather than the MTL in the amyloid-first subtype). We were also limited in our ability to fully characterize the heterogeneity within the tau-first APOE ε4 non-carrier group. Lastly, there were differences in age, education and sex across the ROSMAP and ADNI cohorts that limited our comparisons (Table 1).

In summary, in this study we identified amyloid-first and tau-first patterns of Aβ and tau accumulation using cross-sectional information from *in vivo* and post-mortem data. We found increased Aβ accumulation within the amyloid-first subtype in both ε4 carriers and non-carriers. This supports the idea that both amyloid-first groups belong within the Alzheimer’s disease continuum. Using longitudinal amyloid PET, we found that those in amyloid-first (ε4+) group most likely develop Aβ at an earlier age than those in the amyloid-first (ε4−) group, recapitulating previous findings. Within the tau-first subtype, we found important differences when stratifying by APOE ε4 status. The first is that tau-first ε4 carriers probably belong in the Alzheimer’s disease continuum based on their increased Aβ accumulation, although this group is rare and so has limited longitudinal data. The overwhelming majority of those who develop Alzheimer’s disease are amyloid-first. The second is that tau-first ε4 non-carriers represent PART or are a mixture of PART plus those who accumulate Aβ very slowly. Our findings support the idea that the substantial neocortical tau that is observed in Alzheimer’s disease may result from a local interaction of a slow, age-related tau accumulation process with Aβ. The timing and location of this interaction may be modulated by genetic and modifiable risk factors. These insights into the dynamics of Aβ and tau accumulation may inform research and clinical trials that target these pathologies.

## Supplementary Material

awad232_Supplementary_Data

## Data Availability

ROSMAP data can be requested at: https://www.radc.rush.edu, ADNI data are publicly available at: https://adni.loni.usc.edu and pySuStaIn is freely available at https://github.com/ucl-pond/pySuStaIn. Analysis code used in this study is available upon reasonable request.
