# Supplementary material for: A data-driven study of Alzheimer's disease related amyloid and tau pathology progression
Source: Brain. 2023 Jul 11;146(12):4935–48. doi: 10.1093/brain/awad232 (PMC10690020; doi:10.1093/brain/awad232)
Supplement: awad232_Supplementary_Data [file awad232_supplementary_data.pdf]

Supplementary Table 1

Amyloid PET regions	
Composite SUVR	Component SUVRs
Cingulate	Caudal Anterior Cingulate<br>Rostral Anterior Cingulate<br>Posterior Cingulate<br>Isthmus Cingulate
Inferior Frontal	Pars Opercularis<br>Pars Orbitalis<br>Pars Triangularis
Middle Frontal	Caudal Middle Frontal<br>Rostral Middle Frontal
Superior Frontal	Superior Frontal
Orbitofrontal	Lateral Orbitofrontal<br>Medial Orbitofrontal
Precuneus	Precuneus
Lateral Parietal	Inferior Parietal<br>Superior Parietal<br>Supramarginal<br>Post Central
Lateral Temporal	Inferior Temporal<br>Middle Temporal<br>Superior Temporal<br>Transverse Temporal
Medial Temporal	Entorhinal<br>Parahippocampal<br>Fusiform
Occipital	Cuneus<br>Pericalcarine
**Tau PET regions**	
Composite SUVR	Component SUVRs
Amygdala	Amygdala
Entorhinal	Entorhinal
Medial Temporal	Parahippocampal<br>Fusiform
Lateral Temporal	Inferior Temporal<br>Middle Temporal<br>Superior Temporal<br>Transverse Temporal
Cingulate	Caudal Anterior Cingulate<br>Rostral Anterior Cingulate<br>Posterior Cingulate<br>Isthmus Cingulate
Inferior Frontal	Pars Opercularis<br>Pars Orbitalis<br>Pars Triangularis
Middle Frontal	Caudal Middle Frontal<br>Rostral Middle Frontal
Superior Frontal	Superior Frontal
Orbitofrontal	Lateral Orbitofrontal<br>Medial Orbitofrontal
Lateral Parietal	Inferior Parietal<br>Superior Parietal<br>Supramarginal<br>Post Central
Precuneus	Precuneus
Occipital	Cuneus<br>Lateral Occipital<br>Lingual<br>Pericalcarine

Composite SUVRs used in SuStaIn analysis and their Freesurfer-based component regional SUVRs.

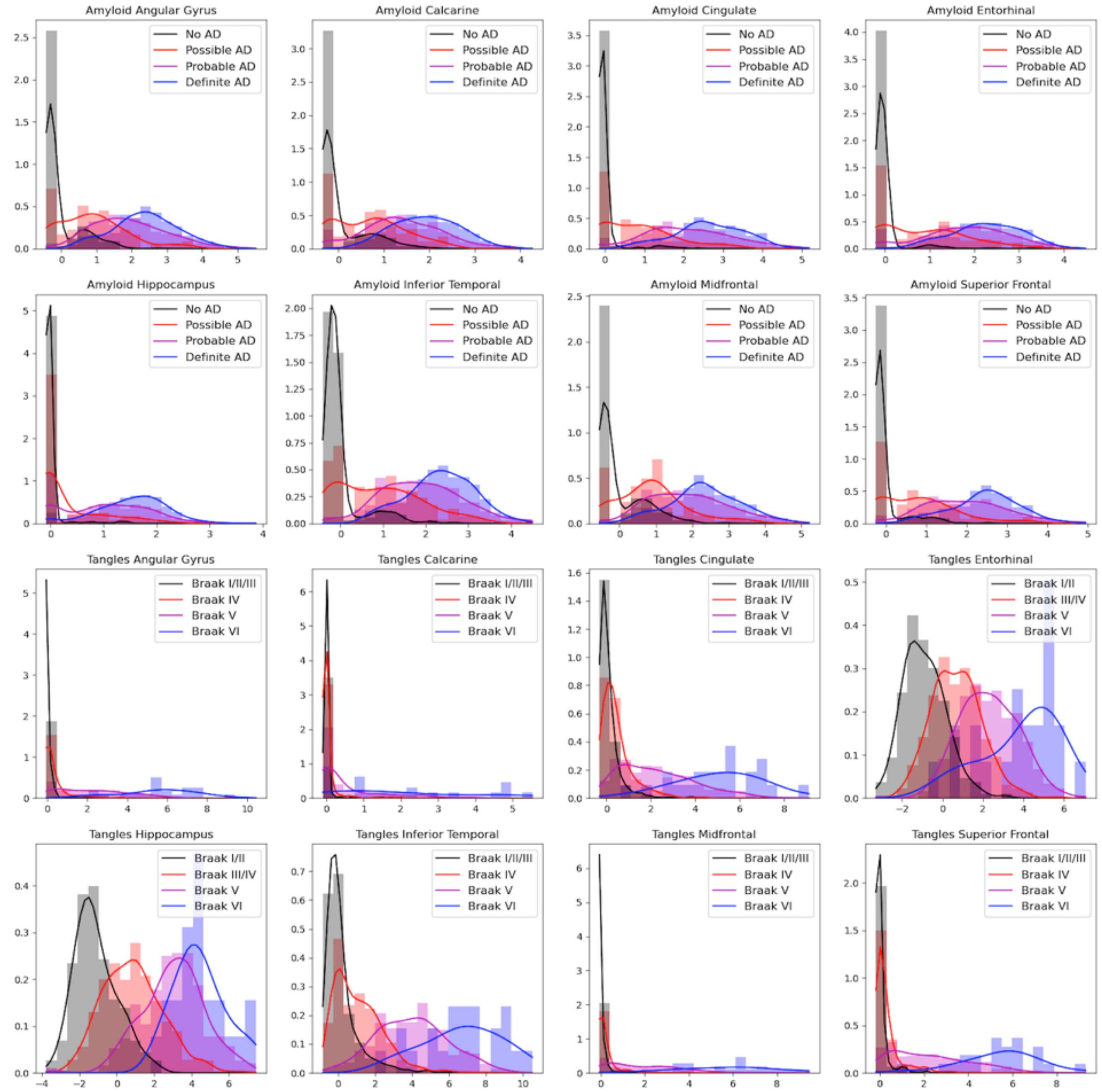

Supplementary Figure 1 Kernel-density estimation based mixture models that were fit for immunohistochemistry-based measures of amyloid beta (percentage area of region occupied by amyloid beta) and tau tangles (cortical density, per mm^2) from the ROSMAP neuropathology study. We took the square root of each measure to improve normality and then residualized each to remove associations with age, sex and education years. For each region we fit a set of mixture models to the residualized amyloid and tau tangle measures using participants' CERAD scores and grouped Braak stages, resulting in set of severity score probabilities for each participant in each region. These probabilities were used as inputs to Ordinal SuStaIn.

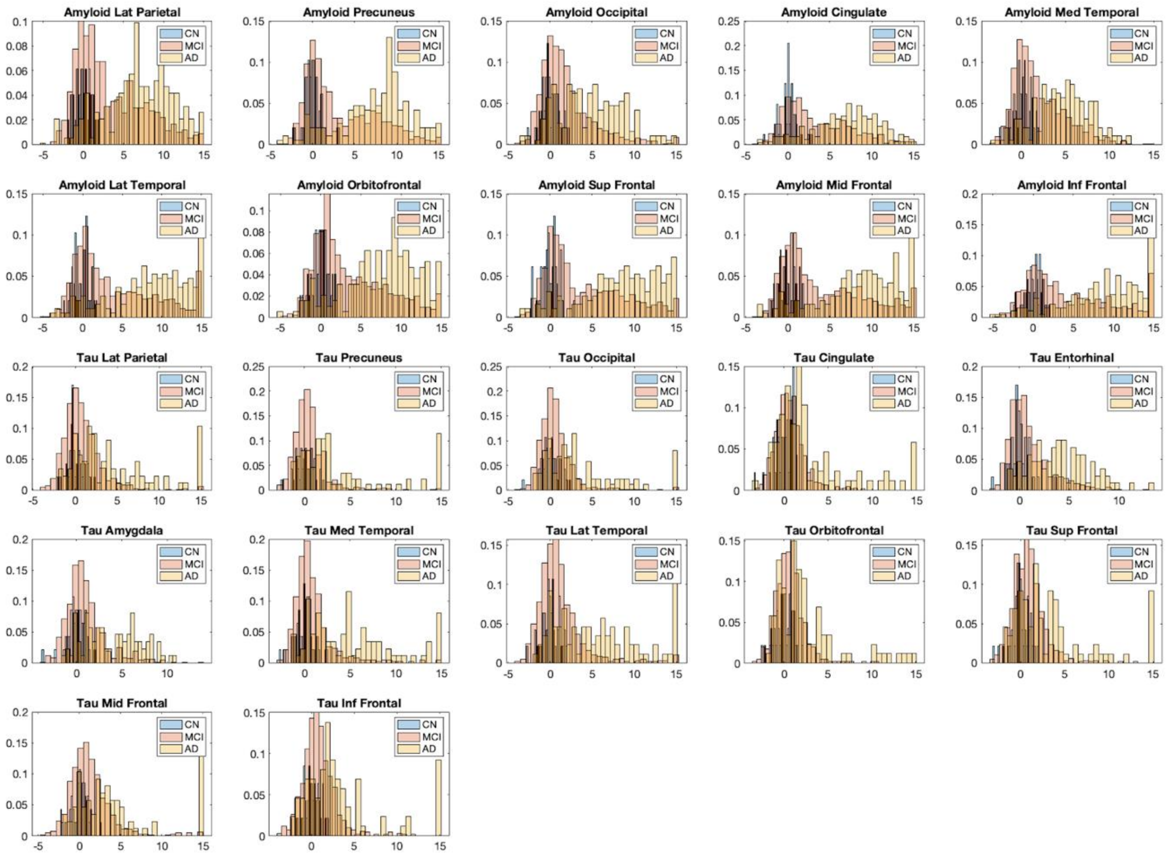

Supplementary Figure 2 Histograms of z-scores for each of the regional SUVRs derived from amyloid PET and tau PET from the ADNI study. Associations with normal aging, sex, education years and intracranial volume were removed prior to z-scoring each marker. CN: cognitively normal; MCI: mild cognitive impairment; AD: Alzheimer’s disease.

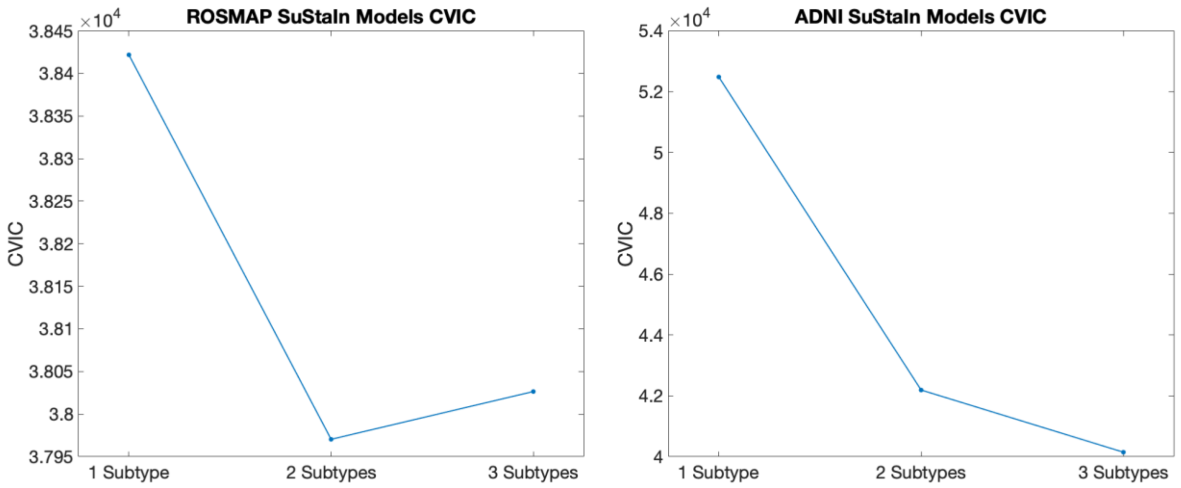

Supplementary Figure 3 Cross-validation information criterion (CVIC) for ROSMAP neuropathology-based analysis and ADNI PET-based analysis.

Supplementary Table 2

Tau PET regions used in longitudinal analysis	
Composite SUVR	Component SUVRs
Braak I	Entorhinal
Braak III/IV	Amygdala<br>Parahippocampal<br>Fusiform<br>Lingual<br>Middle Temporal<br>Inferior Temporal<br>Temporal Pole<br>Caudal Anterior Cingulate<br>Rostral Anterior Cingulate<br>Posterior Cingulate<br>Isthmus Cingulate<br>Insula
Braak V/VI	Superior Frontal<br>Lateral Orbitofrontal<br>Medial Orbitofrontal<br>Frontal Pole<br>Caudal Middle Frontal<br>Rostral Middle Frontal<br>Pars Opercularis<br>Pars Orbitalis<br>Pars Triangularis<br>Lateral Occipital<br>Supramarginal<br>Inferior Parietal<br>Superior Parietal<br>Precuneus<br>Superior Temporal<br>Transverse Temporal<br>Banks of Superior Temporal Sulcus

Composite Braak stage SUVRs used in longitudinal tau PET analysis and their Freesurfer-based component regional SUVRs.

Supplementary Table 3

a. ADNI PET analysis: global amyloid PET SUVR trajectories

	Estimate	SE	t-statistic	P-value
Intercept	0.715	0.048	15.0	$<1\times10^{-6}$
Baseline Age	-0.001	0.000	-3.1	1.90×10^{-3}
Sex (Female)	0.015	0.007	2.2	0.03
Education Years	0.002	0.001	1.8	0.08
ICV	0.000	0.000	1.0	0.32
time	0.000	0.001	-0.7	0.51
Stage 0 (ε4+) intercept	0.003	0.011	0.2	0.82
**A-first (ε4-) intercept**	**0.021**	**0.008**	**2.6**	**8.74×10^{-3}**
**A-first (ε4+) intercept**	**0.047**	**0.008**	**5.7**	**$<1\times10^{-6}$**
T-first (ε4-) intercept	0.008	0.007	1.1	0.25
**T-first (ε4+) intercept**	**0.031**	**0.015**	**2.0**	**0.04**
Stage 0 (ε4+) × time	0.002	0.002	1.0	0.33
**A-first (ε4-) × time**	**0.005**	**0.001**	**3.9**	**8.79×10^{-5}**
**A-first (ε4+) × time**	**0.007**	**0.001**	**5.0**	**$<1\times10^{-6}$**
T-first (ε4-) × time	0.000	0.001	-0.4	0.70
**T-first (ε4+) × time**	**0.008**	**0.002**	**3.4**	**6.26×10^{-4}**

b. ADNI PET analysis: Braak I (entorhinal cortex) tau PET SUVR trajectories

	Estimate	SE	t-statistic	P-value
Intercept	1.005	0.144	7.0	$<1\times10^{-6}$
Baseline Age	0.003	0.001	2.5	0.01
Sex (Female)	-0.017	0.021	-0.8	0.42
Education Years	0.003	0.003	0.9	0.40
ICV	-1.11×10^{-7}	7.45×10^{-8}	-1.5	0.14
time	0.001	0.002	0.4	0.73
Stage 0 (ε4+) intercept	0.018	0.030	0.6	0.54
A-first (ε4-) intercept	0.001	0.023	0.0	0.97
A-first (ε4+) intercept	0.042	0.025	1.7	0.10
**T-first (ε4-) intercept**	**0.062**	**0.023**	**2.7**	**6.65×10^{-3}**
**T-first (ε4+) intercept**	**0.177**	**0.049**	**3.6**	**3.44×10^{-4}**
Stage 0 (ε4+) × time	0.000	0.006	0.0	1.00
A-first (ε4-) × time	0.006	0.004	1.5	0.14
**A-first (ε4+) × time**	**0.020**	**0.005**	**4.1**	**4.71×10^{-5}**
T-first (ε4-) × time	0.005	0.004	1.3	0.20
T-first (ε4+) × time	0.020	0.010	1.9	0.06

c. ADNI PET analysis: Braak III/IV composite tau PET SUVR trajectories

	Estimate	SE	t-statistic	P-value
Intercept	1.192	0.086	13.9	$<1\times10^{-6}$
Baseline Age	-0.001	0.001	-1.1	0.26
Sex (Female)	-0.009	0.013	-0.7	0.48
Education Years	0.001	0.002	0.6	0.57
ICV	-2.56×10^{-8}	4.45×10^{-8}	-0.6	0.57
time	-0.001	0.002	-0.5	0.65
Stage 0 (ε4+) intercept	0.004	0.018	0.2	0.84
A-first (ε4-) intercept	0.014	0.013	1.1	0.28
A-first (ε4+) intercept	0.012	0.015	0.8	0.43
**T-first (ε4-) intercept**	**0.106**	**0.013**	**8.0**	**$<1\times10^{-6}$**
**T-first (ε4+) intercept**	**0.110**	**0.029**	**3.8**	**1.50×10^{-4}**
Stage 0 (ε4+) × time	-0.004	0.004	-0.9	0.35
A-first (ε4-) × time	0.004	0.003	1.2	0.21
**A-first (ε4+) × time**	**0.008**	**0.003**	**2.3**	**0.02**
T-first (ε4-) × time	-0.001	0.003	-0.3	0.76
T-first (ε4+) × time	0.014	0.007	1.8	0.07

d. ADNI PET analysis: Braak V/VI composite tau PET SUVR trajectories

	Estimate	SE	t-statistic	P-value
Intercept	1.168	0.074	15.7	$<1\times10^{-6}$
Baseline Age	-0.002	0.001	-3.7	2.63×10^{-4}
Sex (Female)	0.013	0.011	1.2	0.22
Education Years	0.001	0.002	0.6	0.52
ICV	4.14×10^{-9}	3.86×10^{-8}	0.1	0.91
time	0.001	0.002	0.5	0.63
Stage 0 (ε4+) intercept	0.009	0.015	0.6	0.58
**A-first (ε4-) intercept**	**0.028**	**0.011**	**2.4**	**0.02**
A-first (ε4+) intercept	0.019	0.013	1.5	0.14
**T-first (ε4-) intercept**	**0.118**	**0.011**	**10.4**	**$<1\times10^{-6}$**
**T-first (ε4+) intercept**	**0.105**	**0.025**	**4.2**	**3.18×10^{-5}**
Stage 0 (ε4+) × time	-0.005	0.004	-1.1	0.29
A-first (ε4-) × time	-0.001	0.003	-0.3	0.76
A-first (ε4+) × time	0.002	0.004	0.6	0.55
T-first (ε4-) × time	-0.004	0.003	-1.5	0.12
T-first (ε4+) × time	0.011	0.008	1.5	0.14

Linear mixed effect (LME) models of longitudinal amyloid and tau PET SUVR trajectories. For each model, the stage-zero (APOE ε4-) group is used as the reference group with fixed effects of baseline age (at PET visit), sex, education years, intracranial volume (ICV) at baseline, time (years since baseline), stage-zero (ε4+) group intercept, early A-first (ε4-) group intercept, early A-first (ε4+) group intercept, early T-first (ε4-) group intercept, early T-first (ε4+) group intercept, stage-zero (ε4+) by time interaction, early A-first (ε4-) by time interaction, early A-first (ε4+) by time interaction, early T-first (ε4-) by time interaction, early T-first (ε4+) by time interaction, along with individual-level random intercepts and random slopes with time. For the global amyloid PET SUVR model in **(a)** we used the used the cortical composite reference region, with amyloid positivity threshold of 0.78, which is recommended for longitudinal models (see Methods section). For the tau PET composite SUVR models in **(b)**, **(c)** and **(d)** we used the inferior cerebellum reference region in each case. Supplementary Table 3 lists the composite regions that make up the Braak I SUVR in model **(b)**, the Braak III/IV SUVR in model **(c)** and the Braak V/VI SUVR in model **(d)**.

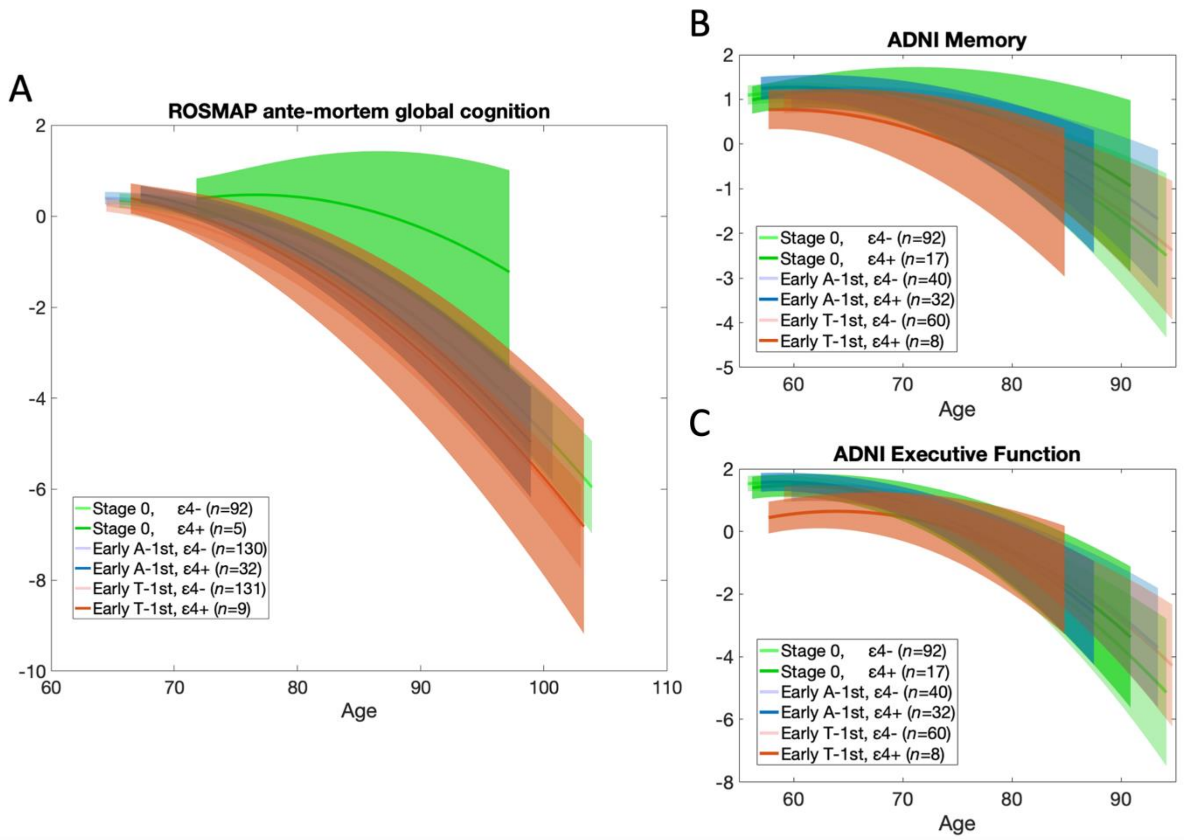

Supplementary Figure 4 Group trajectories of **A** ante-mortem global cognition for participants in ROSMAP neuropathology analysis, showing no differences in baseline levels or rates of decline in global cognitive decline between groups; **B** composite memory in ADNI PET-based analysis, similarly showing no differences and; **C** composite executive function in ADNI, showing a small difference the baseline level of executive function in the 'early tau-first (ε4+)' group relative to the 'stage 0 (ε4)' group but no difference in rates of decline across groups.

Supplementary Table 4

a. ROSMAP neuropathology analysis: ante-mortem global cognition trajectories

	Estimate	SE	t-statistic	P-value
Intercept	0.666	0.337	2.0	0.05
Baseline Age	-0.017	0.004	-4.7	3.18×10^{-6}
Sex (Female)	-0.130	0.055	-2.4	0.02
Education Years	0.048	0.007	6.5	$<1\times10^{-6}$
Time	-0.012	0.011	-1.0	0.30
Time^2	-0.004	0.000	-15.5	$<1\times10^{-6}$
Stage 0 (ε4+)	-0.051	0.227	-0.2	0.82
A-first (ε4-)	0.034	0.069	0.5	0.62
A-first (ε4+)	0.019	0.103	0.2	0.86
T-first (ε4-)	-0.115	0.068	-1.7	0.09
T-first (ε4+)	-0.034	0.173	-0.2	0.84
Stage 0 (ε4+) × time	0.049	0.045	1.1	0.27
A-first (ε4-) × time	0.008	0.014	0.6	0.55
A-first (ε4+) × time	-0.034	0.021	-1.6	0.11
T-first (ε4-) × time	-0.019	0.014	-1.3	0.19
T-first (ε4+) × time	-0.038	0.033	-1.1	0.25

b. ADNI PET analysis: composite memory trajectories

	Estimate	SE	t-statistic	P-value
Intercept	0.526	0.466	1.1	0.26
Baseline Age	-0.013	0.006	-2.3	0.02
Sex (Female)	0.454	0.076	6.0	$<1\times10^{-6}$
Education Years	0.072	0.014	5.1	$<1\times10^{-6}$
Time	0.025	0.011	2.3	0.02
Time^2	-0.003	0.001	-4.1	3.86×10^{-5}
Stage 0 (ε4+) intercept	-0.106	0.153	-0.7	0.49
A-first (ε4-) intercept	-0.069	0.110	-0.6	0.53
A-first (ε4+) intercept	0.165	0.120	1.4	0.17
T-first (ε4-) intercept	-0.075	0.096	-0.8	0.43
T-first (ε4+) intercept	0.060	0.214	0.3	0.78
Stage 0 (ε4+) × time	0.027	0.022	1.2	0.22
A-first (ε4-) × time	0.000	0.016	0.0	0.99
A-first (ε4+) × time	-0.006	0.017	-0.4	0.71
T-first (ε4-) × time	-0.007	0.013	-0.6	0.58
T-first (ε4+) × time	-0.018	0.029	-0.6	0.54

c. ADNI PET analysis: composite executive function trajectories

	Estimate	SE	t-statistic	P-value
Intercept	1.372	0.534	2.6	0.01
Baseline Age	-0.030	0.007	-4.6	3.71×10^{-6}
Sex (Female)	0.164	0.087	1.9	0.06
Education Years	0.102	0.016	6.3	$<1\times10^{-6}$
Time	0.020	0.013	1.6	0.11
Time^2	-0.005	0.001	-5.1	$<1\times10^{-6}$
Stage 0 (ε4+) intercept	-0.119	0.179	-0.7	0.50
A-first (ε4-) intercept	-0.174	0.128	-1.4	0.17
A-first (ε4+) intercept	0.073	0.140	0.5	0.60
T-first (ε4-) intercept	-0.129	0.112	-1.2	0.25
**T-first (ε4+) intercept**	**-0.517**	**0.249**	**-2.1**	**0.04**
Stage 0 (ε4+) × time	0.018	0.023	0.8	0.44
A-first (ε4-) × time	0.002	0.017	0.1	0.89
A-first (ε4+) × time	-0.002	0.018	-0.1	0.90
T-first (ε4-) × time	0.006	0.014	0.4	0.66
T-first (ε4+) × time	0.044	0.029	1.5	0.14

Linear mixed effect (LME) models of (**a**) ante-mortem global cognition using all available longitudinal samples for early-stage participants in the ROSMAP model; (**b**) composite memory and (**c**) composite executive function scores using all available longitudinal samples for early-stage participants in the ADNI model. Each model included fixed effects of baseline age, sex, education years, time (years since baseline), early amyloid-first group intercept (coding for belonging in early amyloid-first group), early tau-first group intercept (same for this group), early-amyloid-first status-by-time interaction and early-tau-fist-by-time interaction, along with individual-level random intercepts and random slopes with time. Supplementary Figure 4 visualizes the predicted trajectories based on these models.

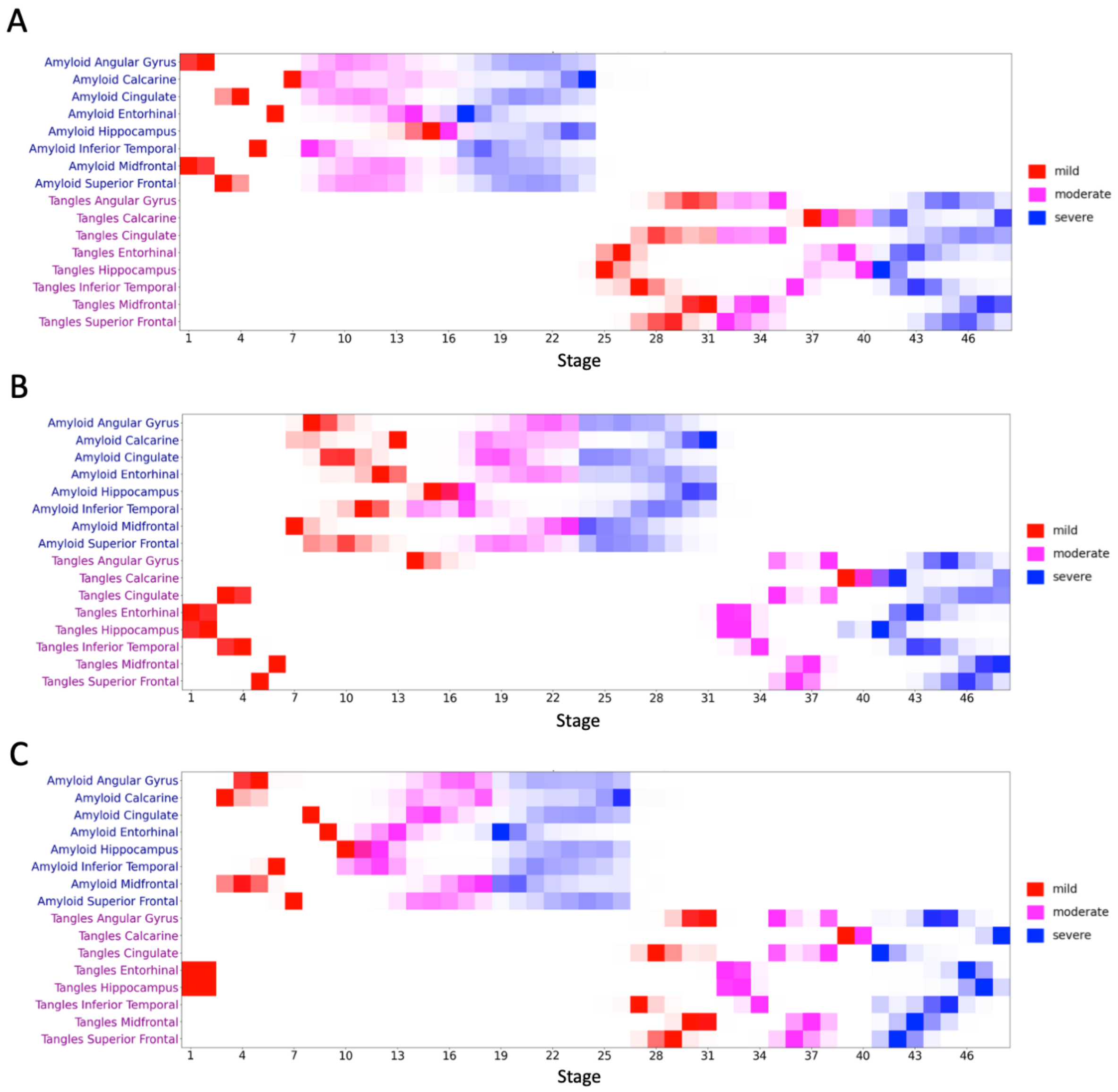

Supplementary Figure 5 A, B, C: Positional variance diagrams (PVDs) for 3-subtype SuStaIn model trained on ROSMAP's neuropathology data. Part **A** is the 'amyloid-first' subtype, **B** is a 'tau-first' with tau tangles in the entorhinal, hippocampal, cingulate, inferior temporal and frontal regions preceding the spread of amyloid, **C** is a second 'tau-first' subtype with early tau tangles restricted to the entorhinal and hippocampal regions.

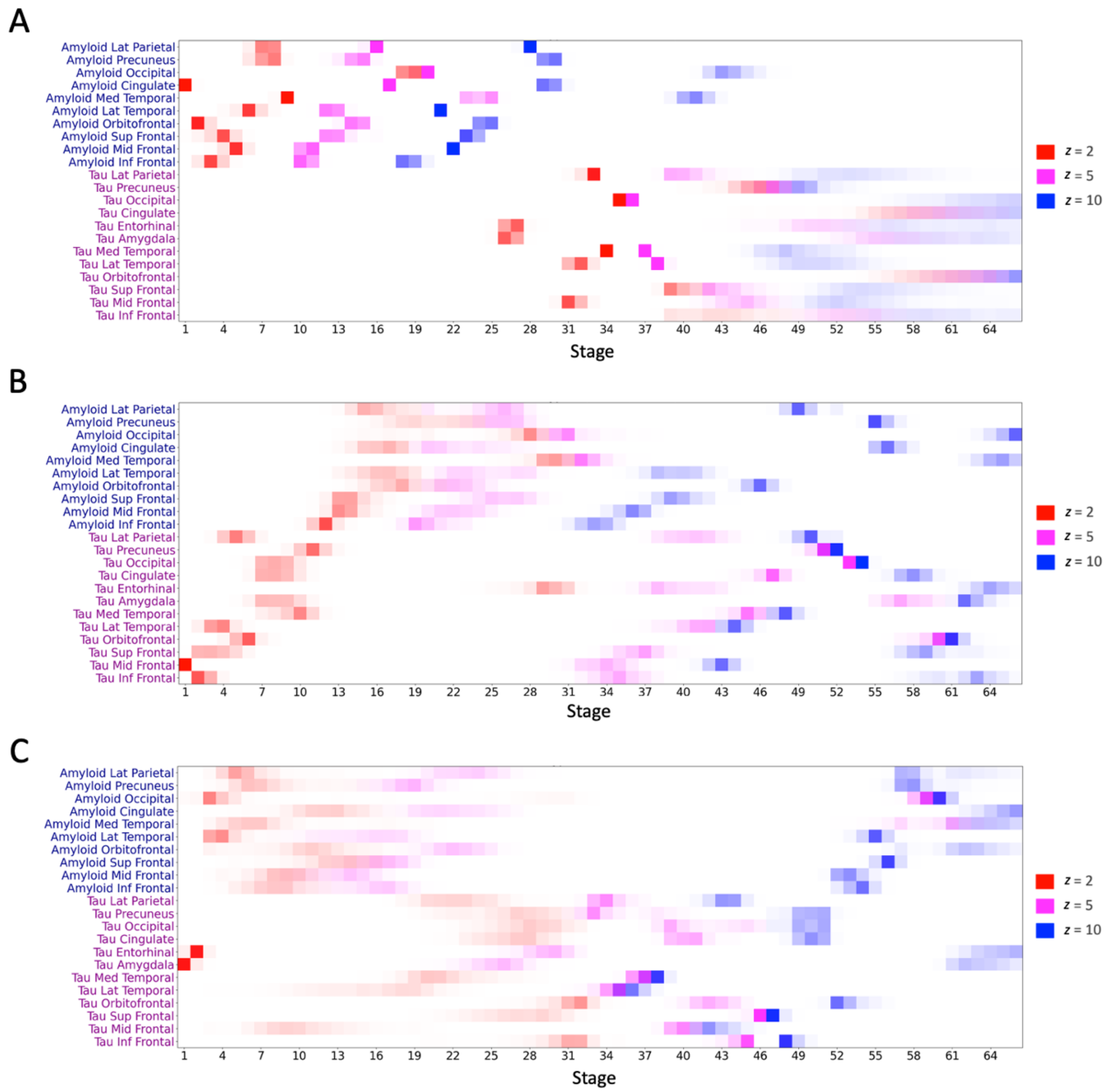

Supplementary Figure 6 A, B, C: Positional variance diagrams (PVDs) for 3-subtype SuStaIn model trained on ADNI's amyloid and tau PET. Part **A** is the 'amyloid-first' subtype, **B** is a 'tau-first' with tau in the entorhinal, hippocampal, cingulate, inferior temporal and frontal regions preceding the spread of amyloid, **C** is a second 'tau-first' subtype with early tau tangles restricted to the entorhinal and hippocampal regions.

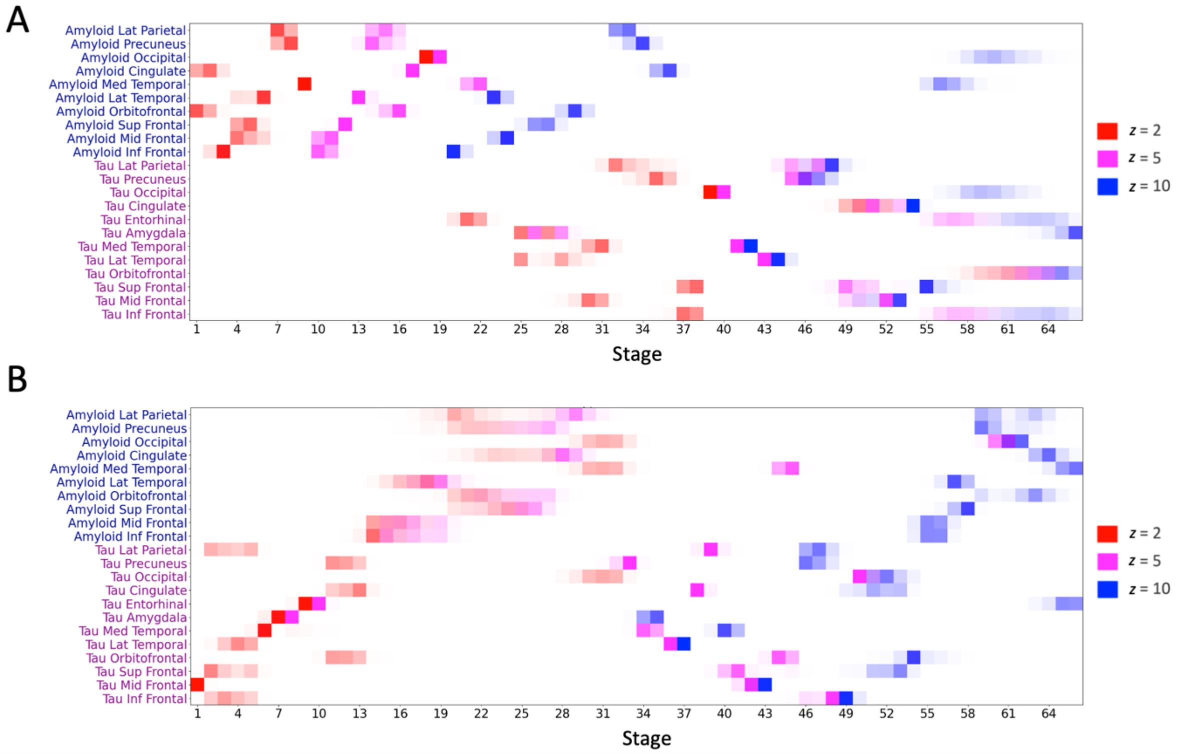

Supplementary Figure 7 A, B: Positional variance diagrams (PVDs) for alternative 2-subtype SuStaIn model trained on ADNI's amyloid PET SUVRs and partial volume corrected tau PET SUVRs. Part **A** is the 'amyloid-first' subtype, **B** is the 'tau-first' subtype.

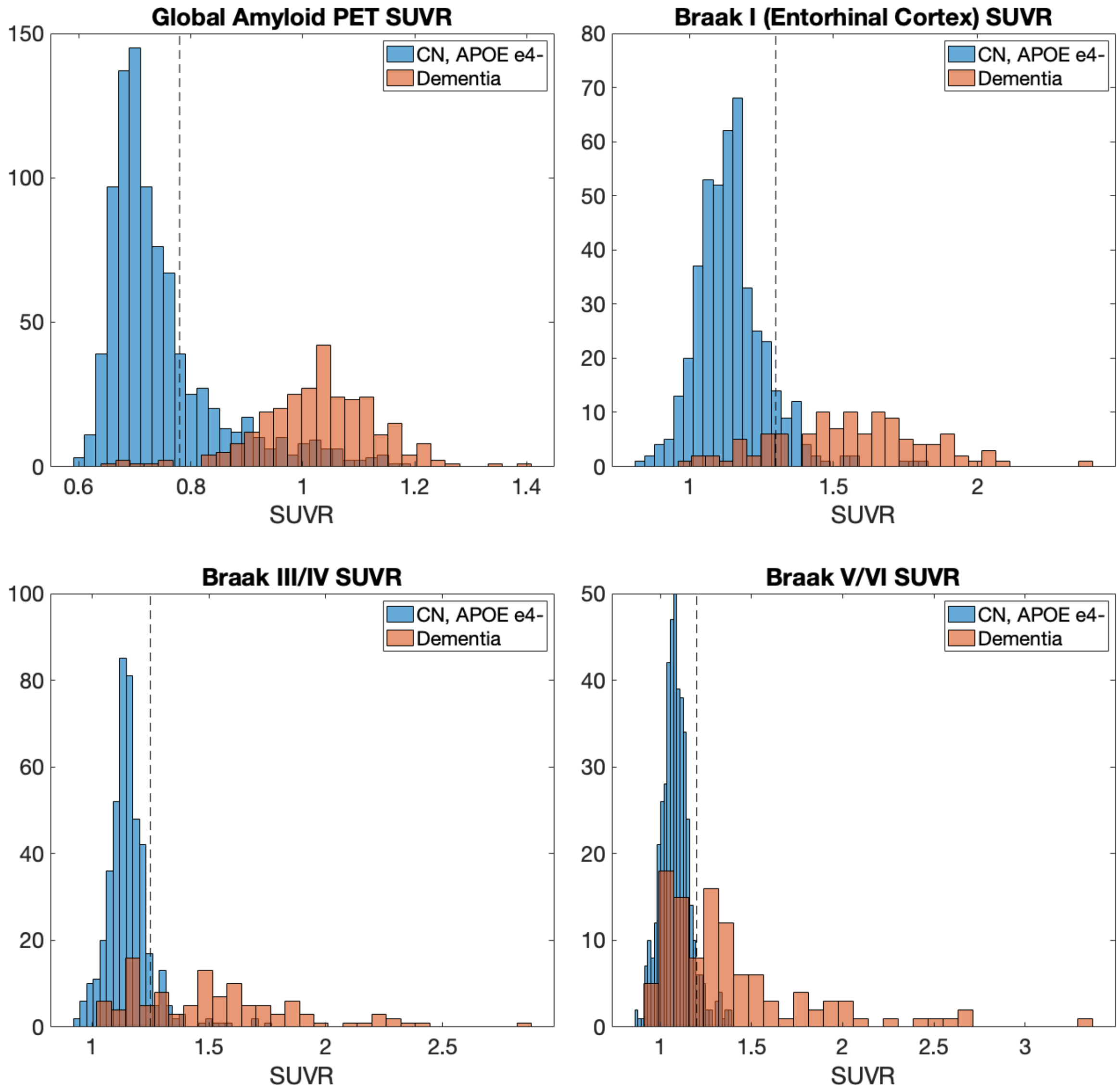

Supplementary Figure 8 Distributions of regional SUVR for cognitively normal (CN), APOE ε4- group and dementia group for **a:** global amyloid PET SUVR showing recommended cut-off of 0.78, **b:** Braak I (entorhinal cortex) composite tau PET SUVR with empirically chosen cut-off of 1.3, **c:** Braak III/IV composite tau PET SUVR region with empirically chosen cut-off of 1.25, d: Braak V/VI composite tau PET SUVR with empirically chosen cut-off of 1.2. Component regions for each Braak composite region are listed in Supplementary Table 2.
